# Comparison of shear bond strength of new and rebonded ceramic brackets with and without hydrofluoric acid conditioning: an in vitro study

**DOI:** 10.1186/s12903-026-07797-7

**Published:** 2026-02-07

**Authors:** Aqlan Ali Moqbel Qaid Al-Kamel, Shirchie Iris P. Galvan, Galahad T. Perea, Salem Omar Bin Jahlan, Aisha Ghazi Yahya

**Affiliations:** 1 MCU -Department of Orthodontics, College of Dentistry, Manila Central University, Caloocan, Philippines; 2 Manila Central University, Caloocan, Philippines; 3 IBB University, Ibb, Yemen

**Keywords:** Ceramic brackets, Monocrystalline brackets, Shear bond strength, Hydrofluoric acid, Airborne particle abrasion, Rebonding ceramic brackets

## Abstract

**Background:**

Ceramic orthodontic brackets are widely used for their aesthetic advantages; however, their bonding strength, particularly upon rebonding, remains a concern. Effective surface-conditioning methods are essential to optimize shear bond strength (SBS) under laboratory conditions. Hydrofluoric acid (HF) conditioning and airborne particle abrasion (APA) are commonly employed techniques to enhance bracket adhesion.

**Methods:**

Thirty monocrystalline ceramic brackets were randomly allocated into three groups (*n* = 10 each). Group A (control) consisted of new ceramic brackets; Group B included rebonded brackets treated with tungsten carbide burs, APA for ≥ 30 s, and 9.5% HF for 90 s; Group C included rebonded brackets treated with tungsten carbide burs and APA only. SBS was measured using a universal testing machine at 0.5 mm/min until bracket detachment, and values were recorded in MPa.

**Results:**

The mean SBS for new brackets was 13.51 ± 5.61 MPa. Rebonded brackets with HF conditioning (Group B) showed 6.88 ± 2.55 MPa, and rebonded brackets without HF conditioning (Group C) showed 6.36 ± 3.60 MPa. One-way ANOVA revealed a statistically significant difference among groups (*p* = 0.0287). Tukey’s post hoc analysis indicated significantly higher SBS for Group A compared with Group B (*p* = 0.007) and Group C (*p* = 0.011), as well as a significant difference between Groups B and C (*p* = 0.009).

**Conclusions:**

New ceramic brackets demonstrated significantly higher immediate SBS than rebonded brackets under in vitro conditions. Although HF conditioning slightly enhanced immediate SBS compared with APA alone, both methods yielded lower bond strengths than new brackets. Within the limitations of this in vitro study, APA may be considered a practical alternative to HF conditioning for rebonded ceramic brackets.

**Supplementary Information:**

The online version contains supplementary material available at 10.1186/s12903-026-07797-7.

## Background

Orthodontic treatment plays a fundamental role in improving both dental function and facial esthetics. Among the various fixed appliance options, ceramic brackets have become increasingly popular due to their translucent, tooth-colored appearance, offering superior esthetic appeal to patients [1]. In particular, monocrystalline alumina brackets are valued for their high translucency and adequate mechanical strength. Despite these advantages, bond failure at the enamel adhesive bracket interface remains a common clinical challenge during orthodontic treatment [2, 3]. Such failure can result from factors such as inadequate adhesion, bracket design limitations, or accidental debonding caused by occlusal forces and patient habits. In clinical practice, recycling or rebonding debonded ceramic brackets is often considered, especially in cost-sensitive settings or when replacement is not immediately feasible [4]. However, rebonding typically results in reduced shear bond strength (SBS) compared to new brackets [5], raising concerns about the long-term stability of the adhesive interface. Maintaining adequate SBS is crucial for the success of fixed orthodontic treatment, as insufficient bonding may lead to prolonged treatment time, patient discomfort, and increased clinical workload.

Several mechanical and chemical surface treatments have been investigated to restore SBS in rebonded brackets. These include tungsten carbide bur abrasion, airborne particle abrasion (APA), hydrofluoric acid (HF) etching, universal primers, and laser-based approaches [6–12]. Comparisons between HF and APA ± silane treatments have shown that APA with silane can achieve SBS levels meeting or exceeding clinical acceptability, while some laser protocols, such as Er: YAG, can produce SBS and Adhesive Remnant Index (ARI) results comparable to new brackets without damaging the bracket base [13, 14].

Despite extensive research, the potential implications of hydrofluoric acid (HF) use during rebonding remain insufficiently defined in vitro. Although alumina is highly resistant to acid etching, HF may create superficial micro pitting and increase surface energy, thereby enhancing micromechanical retention between the bracket and adhesive. Limited comparative evidence exists specifically for monocrystalline ceramic brackets under standardized adhesive removal and conditioning protocols. Therefore, this study focused on comparing the shear bond strength (SBS) of new and rebonded brackets with and without HF treatment under identical laboratory conditions.

In this study, effectiveness refers to the ability of a surface conditioning method to improve the SBS of rebonded monocrystalline ceramic brackets relative to untreated rebonded surfaces, while maintaining bracket base integrity. This in vitro study aims to compare the immediate shear bond strength of new and rebonded monocrystalline ceramic brackets after adhesive removal with a tungsten carbide bur, followed by either HF conditioning or no HF conditioning, under standardized conditions. The null hypothesis stated that there would be no statistically significant difference in shear bond strength between new and rebonded monocrystalline ceramic brackets treated with or without hydrofluoric acid conditioning.

## Methods

### Research design

This comparative in vitro experimental study evaluated the shear bond strength (SBS) of monocrystalline ceramic orthodontic brackets subjected to three different surface-conditioning protocols. The specimens were randomly allocated into three experimental groups (*n* = 10 per group) as follows:

- Group A (Control – New brackets): New ceramic brackets bonded without any surface treatment.
- Group B (Rebonded – Tungsten carbide + airborne particle abrasion + HF): Bracket bases were conditioned using a micro-blaster with 50 µm aluminum oxide particles at 90 psi, a working distance of 12 mm, and a 45° angulation for a minimum of 30 seconds, followed by conditioning with 9.5% hydrofluoric acid (HF) for 90 seconds. A silane primer was applied prior to rebonding.
- Group C (Rebonded – Tungsten carbide + airborne particle abrasion only): The same procedure as Group B was applied, except that HF conditioning and silane application were omitted.The dependent variable was shear bond strength (MPa), while the independent variable was the surface-treatment protocol with three levels corresponding to the experimental groups.

### Specimen preparation

Thirty recently extracted human premolar teeth (*n* = 30), indicated for orthodontic treatment, were collected for this study. Teeth were selected based on the presence of intact buccal enamel surfaces free from cracks, caries, restorations, or visible defects. Teeth exhibiting developmental anomalies, fluorosis, enamel hypoplasia, carious lesions, or signs of external root resorption were excluded. External root resorption was assessed under ×3.5 magnification. Immediately after extraction, all specimens were cleaned of residual soft tissue using a hand scaler and disinfected in 0.5% chloramine-T solution for 24 hours. The teeth were subsequently stored in deionized water at 4 °C, with weekly replacement of the storage medium, until further experimental procedures.

### Sample size calculation

The required sample size was calculated using the formula for one-way analysis of variance (ANOVA) described by Liu and Wang [15]:

$$\mathrm{n}=\left[\left(\mathrm{Z}\upalpha/2+\mathrm{Z}\upbeta\right)^2\times2\times\;\left(\upsigma^2\right)\right]\div\;\left(\mathrm{d}^2\right)$$

where:

- Zα/2 = 1.96 for a 95% confidence level;
- Zβ = 0.84 for 80% statistical power;
- d = 5 MPa, representing the expected difference between group means derived from Yousef et al. [7] and Sajitha et al. [16].

The calculation indicated a minimum of nine specimens per group. To ensure adequate statistical power and account for potential specimen loss, ten specimens were included in each group.

### Materials and instruments

The materials used in this study, along with their composition, brand names, and manufacturers, are listed in Table 1.

**Table 1:** Materials used in the study and their composition

Material	Composition	Brand/Model	Manufacturer
Monocrystalline ceramic brackets (Roth prescription, 0.022-inch slot)	Single-crystal aluminum oxide (sapphire)	*Radiance™*	American Orthodontics, Sheboygan, WI, USA
Hydrofluoric acid etchant	9.5% hydrofluoric acid gel	*Porcelain Etchant*	Bisco, Schaumburg, IL, USA
Silane primer	Organosilane-based primer	*Porcelain Primer*	Bisco, Schaumburg, IL, USA
Phosphoric acid etchant	37% phosphoric acid gel	*Etch-37™*	Bisco, Schaumburg, IL, USA
Light-cured orthodontic adhesive resin	Bis-GMA–based composite resin	*Transbond™ XT*	3 M Unitek, Monrovia, CA, USA
Light-cured orthodontic adhesive primer	Unfilled resin-based primer	Transbond™ XT Primer	3 M Unitek, Monrovia, CA, USA
Disinfectant/storage solution	0.5% chloramine-T solution	Chloramine-T	Merck KGaA, Darmstadt, Germany
Self-cure acrylic resin	Polymethyl methacrylate–based resin	Orthoresin^®^	Dentsply Sirona, York, PA, USA
Aluminum oxide particles for APA	50 μm Al2O3	Aluminum oxide powder	Renfert GmbH, Hilzingen, Germany

### Teeth selection, storage and mounting

Thirty freshly extracted human premolars indicated for orthodontic treatment were selected and randomly allocated into three experimental groups (*n* = 10 per group). Inclusion criteria comprised fully developed roots, intact buccal enamel, and the absence of caries, fractures, hypoplasia, discoloration, or previous chemical treatment. Teeth exhibiting external root resorption (assessed visually under ×3.5 magnification), damage during extraction, or storage exceeding three months were excluded.

Immediately after extraction, all teeth were disinfected in 0.5% chloramine-T solution for 24 h and subsequently stored in the same solution at 4 °C until use, following the protocol described by Boruziniat et al. [17]. Prior to mounting, the teeth were rinsed with deionized water and air-dried.

Each specimen was embedded vertically in self-cure acrylic resin (Orthoresin^®^, Dentsply, USA) within a custom-made cylindrical metal mold (30 mm in height × 20 mm in diameter). Each tooth was positioned centrally with 2 mm of the crown exposed cervically from the cementoenamel junction (CEJ). The specimens were embedded to achieve a vertical orientation with the labial surface perpendicular to the direction of the applied shear load.

After polymerization, the acrylic blocks were color-coded according to the experimental groups (Group A: pink; Group B: orange; Group C: blue) to facilitate identification during testing. All specimens were mounted in the universal testing machine with the labial surface maintained perpendicular to the direction of force application.

### Bonding, debonding, conditioning, and rebonding protocols

#### Bonding Protocol (initial bonding for all groups)

Group A (Control – New brackets): The buccal enamel surface of each specimen was cleaned using a fluoride-free pumice slurry. The enamel was etched with 37% phosphoric acid gel for 30 s, rinsed with water for 15 s, and dried with oil-free compressed air until a chalky white appearance was obtained. A thin layer of Transbond™ XT primer was applied to the etched enamel surface and the bracket base. The brackets were bonded using Transbond™ XT adhesive (3 M Unitek, Monrovia, CA, USA) and positioned on the middle third of the buccal surface. Each bracket was light-cured for 30 s. The bonded specimens were stored in deionized water at 37 °C for 24 h prior to further procedures [6, 9].

Groups B and C (Rebonded brackets – Initial Bonding): For the initial bonding phase of Groups B and C, the same adhesive system (Transbond™ XT primer and adhesive; 3 M Unitek, Monrovia, CA, USA) was used. In these groups, phosphoric acid etching of the enamel surface was not performed. The brackets were positioned on the buccal surface and light-cured for 30 s. All specimens were stored in deionized water at 37 °C for 24 h prior to the debonding procedure [6, 9].

#### Debonding and conditioning protocol

After 24 h of storage, the brackets in Groups B and C were debonded using orthodontic pliers by applying pressure to the bracket wings. Each bracket was examined under ×3.5 magnification and dental light to ensure integrity, and any fractured brackets were discarded and replaced to maintain specimen consistency.

- Group A (Control – New brackets): Brackets in this group were not subjected to any surface treatment and served as the baseline for comparison.
- Group B (Rebonded – TC + APA + HF): For rebonded specimens, residual adhesive was removed using a tungsten carbide (TC) bur at 25,000 rpm under continuous water cooling. The bracket bases were then subjected to APA with 50 µm Al₂O₃ particles at 620 kPa pressure, a 12 mm working distance, and a 45° angulation, for a minimum of 30 seconds until a uniform frosted surface was obtained. After rinsing with water and air-drying, the bracket base was conditioned with 9.5% hydrofluoric acid (HF) applied via sponge pad for 90 seconds, as recommended by the manufacturer (Bisco, Schaumburg, IL, USA). This HF conditioning time and concentration were adapted from Sajitha et al. [16], who used 9.6% HF for 90 seconds when reconditioning ceramic brackets. The samples were then rinsed with water for 15 seconds, dried with oil-free air, and a silane primer was applied for 30 seconds and air dried according to the manufacturer’s instructions, without additional light-curing, before rebonding[6, 9,16].
- Group C (Rebonded – TC + APA only): The same procedure described for Group B was followed, except that hydrofluoric acid conditioning and silane application were omitted. After airborne particle abrasion, the bracket bases were rinsed thoroughly with water and dried with oil-free air prior to rebonding [9, 18].

**Fig. 1:**
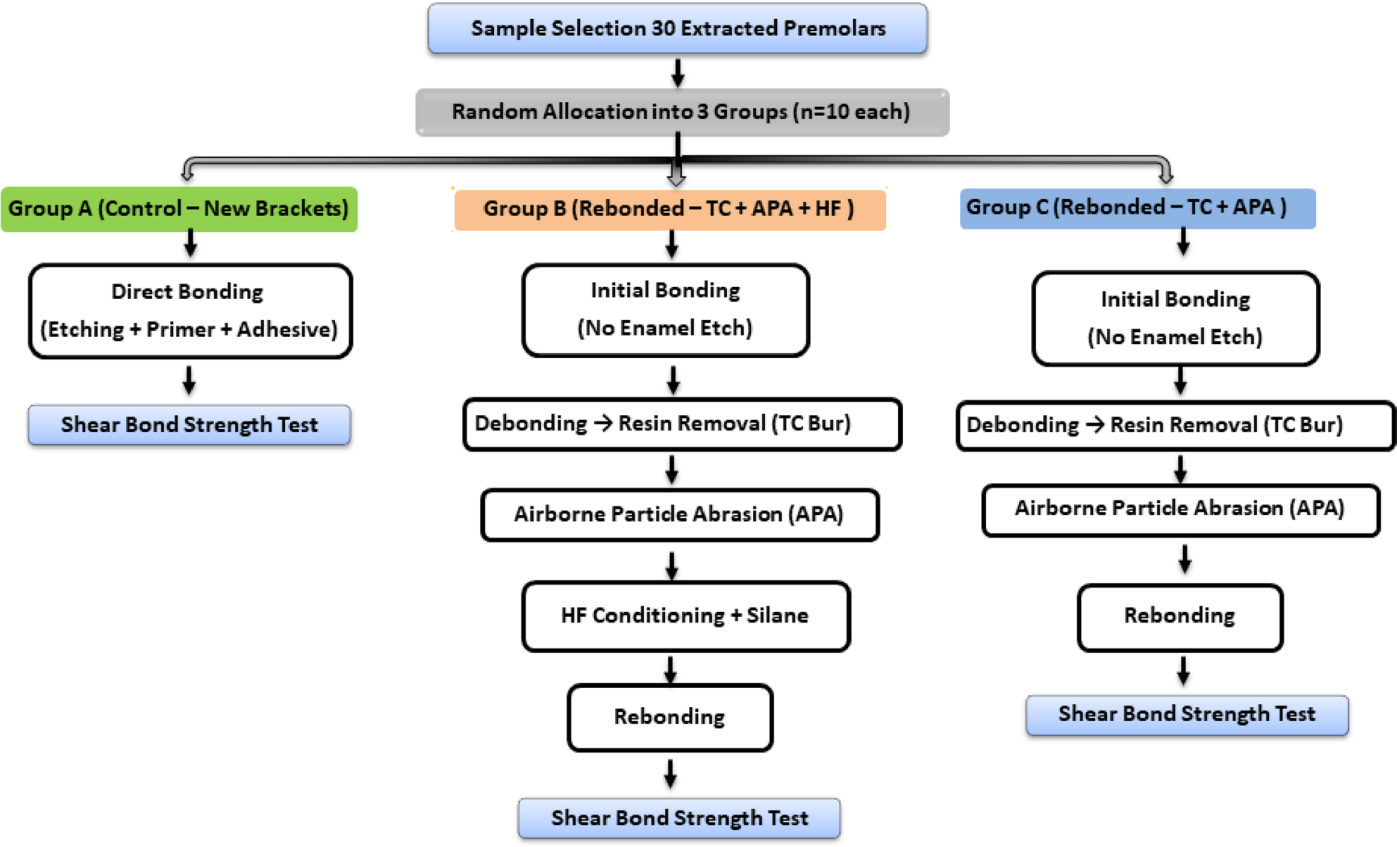
Flowchart of study methodology

The experimental groups and surface-conditioning protocols are summarized in Table 2, and the sequential steps of the methodology are illustrated in Fig. 1.

**Table 2:** Summary of experimental groups and surface-conditioning protocols

Group	Bracket Type	Conditioning Protocol	Notes
A (Control)	New monocrystalline ceramic brackets	No surface treatment (direct bonding only)	Baseline group
B	Rebonded monocrystalline ceramic brackets	TC bur (25,000 rpm, water cooling) + (APA; 50 μm Al₂O₃, 620 kPa, 12 mm, 45°, minimum 30 s) + 9.5% HF (90 s) + Silane (30 s, air-dried)	Full surface conditioning
C	Rebonded monocrystalline ceramic brackets	TC bur (25,000 rpm, water cooling) + APA (50 μm Al₂O₃, 620 kPa, 12 mm, 45°, minimum 30 s) only	Without HF and Silane

Schematic representation of the experimental workflow illustrating sample preparation, bonding, debonding, surface conditioning, rebonding, and shear bond strength (SBS) testing

**Fig. 2:**
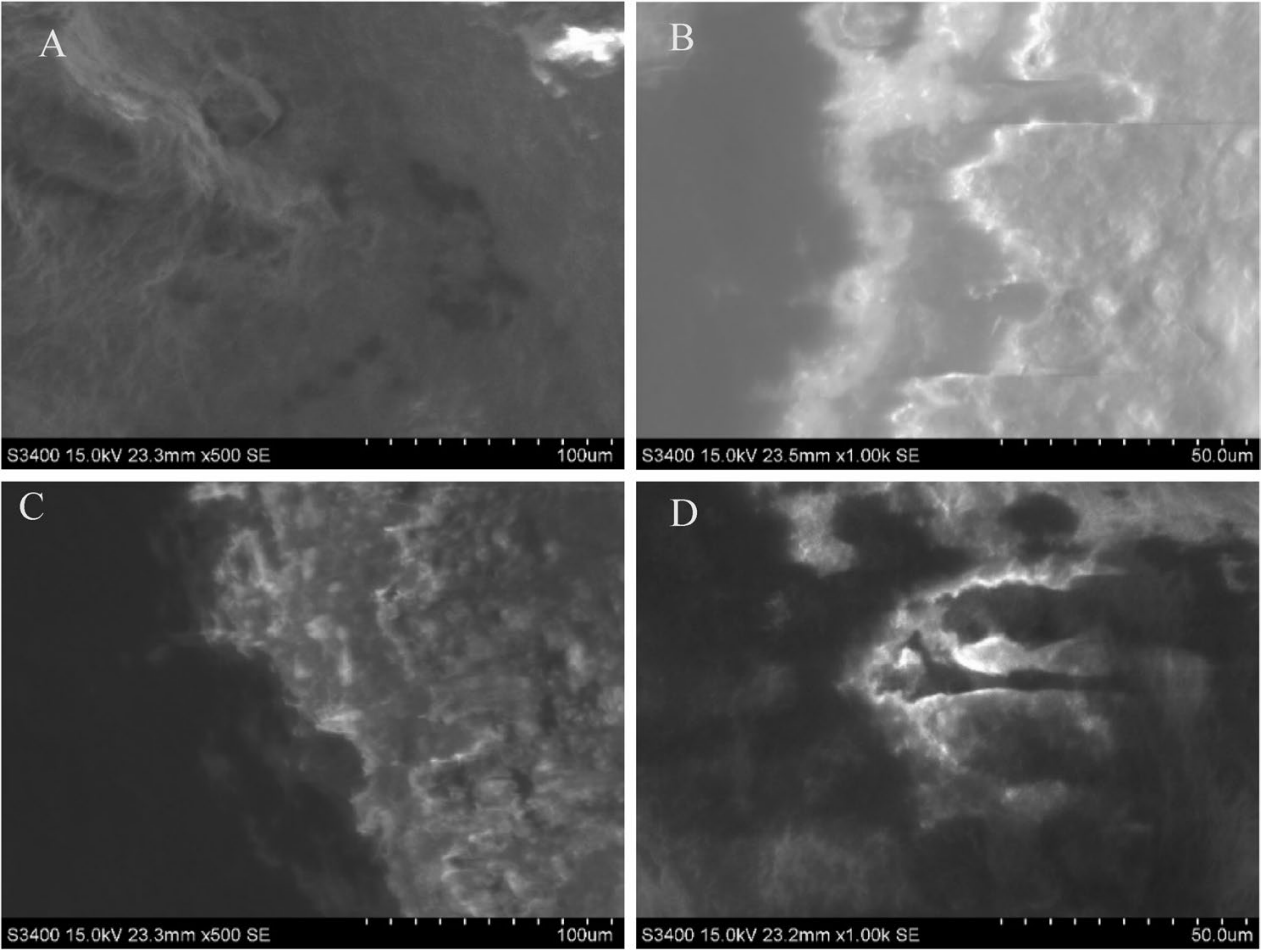
SEM images of bracket-base surfaces at 500× and 1000× magnifications. **A**–**B** Group **B** (APA + HF); (**C**–**D**) Group C (APA only)

A preliminary pilot test was performed prior to the main experiment to validate the adequacy of the adhesive-removal and surface-conditioning protocols. Six monocrystalline ceramic brackets from each rebonded group were examined under a scanning electron microscope (SEM) at 500× and 1000× magnifications. Group B (treated with HF) exhibited roughened, etched surfaces with distinct micro-pits, whereas Group C (without HF) displayed smoother surfaces with minor adhesive remnants. These qualitative observations verified the effectiveness and integrity of the surface-conditioning procedures adopted for the main experiment. Representative SEM images are presented in Fig. 2.

### Rebonding protocol

Rebonding was performed using a conventional bonding technique described by Kilinc and Sayar (2019) [19]. After completing the surface-conditioning procedures detailed previously, the treated brackets in Groups B and C were rebonded onto their respective teeth using the same adhesive system and curing parameters as in the initial bonding phase to ensure procedural consistency. The buccal enamel surface was etched with 37% phosphoric acid for 30 s, rinsed thoroughly with water, and dried with oil-free air. A thin layer of Transbond™ XT primer was applied to the enamel surface, followed by Transbond™ XT adhesive (3 M Unitek, Monrovia, CA, USA) on the bracket base. Each bracket was positioned accurately on the middle third of the buccal surface and light-cured for 30 s. All rebonded specimens were stored in deionized water at 37 °C for 24 h before shear bond strength (SBS) testing [6, 9].

### Shear bond test

**Fig. 3:**
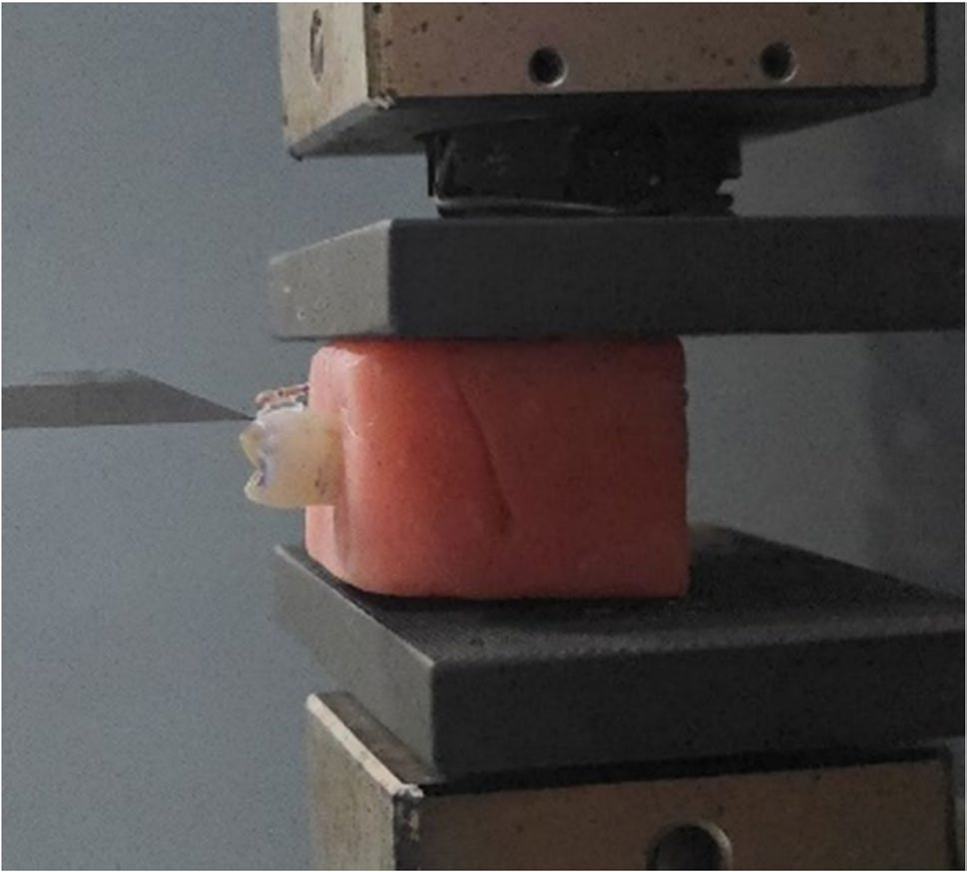
Shear bond strength testing setup

The shear bond strength (SBS) of each specimen was evaluated using a universal testing machine (Instron^®^, 100 kN; Instron Corp., Norwood, MA, USA). Each acrylic block was mounted in a custom metal jig to ensure that the bracket base was parallel to the direction of the applied shear force. A chisel-edged plunger attached to the crosshead applied a controlled shear load at a crosshead speed of 0.5 mm/min until bracket debonding occurred (Fig. 3). The load at failure (N) was automatically recorded by the testing software. Shear bond strength was calculated in megapascals (MPa) using the following equation:

$$SBS\left(MP\upalpha\right)\;={Force}\left(N\right)\;/Bracket base area\left(mm^2\right)$$

A constant bracket-base area of 15.08 mm², as specified by the manufacturer (American Orthodontics, USA), was used for all specimens to maintain dimensional uniformity and ensure intergroup comparability, in accordance with Yousef et al. [7] and Sajitha et al. [16]. All bonding and testing procedures were carried out under standardized environmental conditions (23 ± 1 °C and 50 ± 5% relative humidity).

Thermocycling and artificial aging were intentionally omitted to isolate and compare the immediate effects of different surface-conditioning methods on shear bond strength. This approach followed previously published in vitro studies on monocrystalline ceramic brackets [7, 16], which used the same experimental conditions to assess the influence of hydrofluoric acid and airborne particle abrasion on bond performance.

A chisel-edged plunger applying controlled shear force at a crosshead speed of 0.5 mm/min to debond ceramic brackets from the tooth surface.

### Statistical analysis

Statistical analyses were performed using IBM SPSS Statistics for Windows, Version 26.0 (IBM Corp., Armonk, NY, USA). The dependent variable was the shear bond strength (SBS, MPa), and the independent variable was the surface-conditioning method (three levels: Group A, Group B, and Group C). Data normality was assessed using the Shapiro–Wilk test, and the homogeneity of variances was verified using Levene’s test. Since all assumptions for parametric testing were satisfied, a one-way analysis of variance (ANOVA) was performed to compare mean SBS values among the groups, followed by Tukey’s post hoc test for multiple pairwise comparisons. Descriptive data are presented as mean ± standard deviation (SD), and a *p*-value < 0.05 was considered statistically significant [15].

## Results

### Shear bond strength (descriptive statistics)

**Fig. 4:**
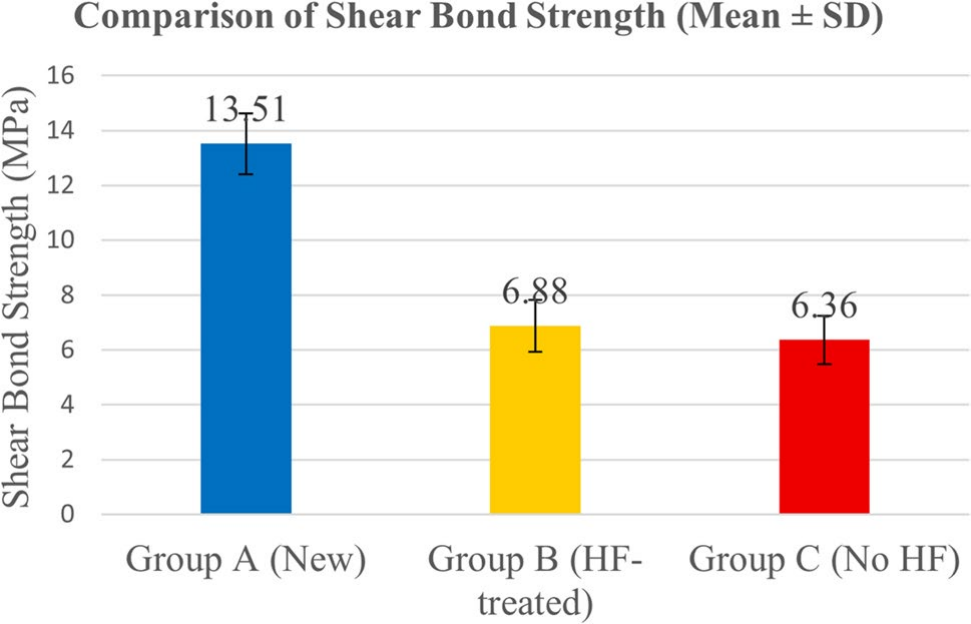
Mean SBS values (MPa) with standard deviations for Groups A, B, and C

The descriptive statistics of shear bond strength (SBS) for the three experimental groups are presented in Table 3. Group A (new brackets) exhibited the highest mean SBS value (13.51 ± 5.61ᵃ MPa), whereas the rebonded groups showed notably lower mean values. Group B (HF-treated) recorded 6.88 ± 2.55ᵇ MPa, and Group C (no HF treatment) recorded 6.36 ± 3.60^c^ MPa. Mean SBS values with corresponding standard deviations are illustrated in Fig. 4.

**Table 3:** Descriptive statistics of shear bond strength (SBS) values (MPa)

Group	*N*	Mean ± SD	Minimum	Maximum
A (New brackets)	10	13.51 ± 5.61ᵃ	2.45	21.81
B (Rebonded with HF)	10	6.88 ± 2.55ᵇ	1.85	11.32
C (Rebonded without HF)	10	6.36 ± 3.60^c^	1.92	15.10

Different superscript letters (a, b, c) indicate statistically significant differences among groups (*p* < 0.05, Tukey’s HSD post hoc test)

### Normality and ANOVA results

The Shapiro–Wilk test confirmed that the SBS data were normally distributed across all groups (*p* > 0.05). Levene’s test also verified homogeneity of variances (*p* > 0.05). A one-way analysis of variance (ANOVA) revealed a statistically significant difference in SBS among the three groups (*p* = 0.0287). Post hoc comparisons using Tukey’s test indicated that Group A (new brackets) exhibited significantly higher SBS than both Group B (HF-treated) and Group C (no HF), with all pairwise comparisons reaching statistical significance (*p* < 0.05).

### Post hoc pairwise comparisons

Tukey’s post hoc analysis demonstrated significant differences between all groups (Table 4). The mean SBS of new brackets (Group A) was significantly higher than that of HF-treated rebonded brackets (Group B) (*p* = 0.007) and non-HF rebonded brackets (Group C) (*p* = 0.011). Moreover, a smaller but statistically significant difference was found between Groups B and C (*p* = 0.009), indicating that HF treatment produced a modest yet measurable improvement in the surface bonding efficiency of rebonded brackets.

**Table 4:** Tukey’s post hoc pairwise comparisons for Shear Bond Strength (SBS)

Comparison	*p*-value	Interpretation
A vs. B	0.007	Significant
A vs. C	0.011	Significant
B vs. C	0.009	Significant

## Discussion

The present study compared the shear bond strength (SBS) of new and rebonded monocrystalline ceramic brackets subjected to different surface-conditioning protocols. The null hypothesis stating that no significant difference exists among the groups was rejected, as statistically significant differences were observed between new and rebonded brackets and also between the two rebonded groups, indicating that surface-conditioning methods influence bonding outcomes.

New brackets demonstrated the highest SBS values, which is consistent with previous studies reporting superior adhesion of unused ceramic brackets compared with recycled ones [5, 16]. This finding is commonly attributed to the intact bracket-base topography of new brackets, which favors micromechanical retention and more predictable bonding. Sajitha et al. (2020) [16] similarly reported that new monocrystalline ceramic brackets achieve reliable bond strength while reducing the risk of enamel damage during debonding.

Among rebonded brackets, the HF-treated group (Group B) exhibited moderately higher SBS values than the APA only group (Group C). This difference may be related to the combined effects of mechanical surface roughening and chemical surface modification. Although alumina-based ceramics are resistant to deep acid dissolution, previous studies have reported that hydrofluoric acid (HF) can induce superficial surface irregularities and increase surface energy, which may enhance wettability and micromechanical interaction with resin adhesives [9, 16]. This effect could account for the slightly higher SBS values observed in the HF-treated group compared with APA alone.

Nevertheless, the conditioning effect of HF remains less pronounced than that achieved by APA combined with silane treatment [6, 7, 10]. Silane coupling agents further promote chemical bonding through siloxane linkages, reinforcing the bracket adhesive interface [6]. SEM findings from the pilot validation in this study support these mechanisms, as HF-treated brackets exhibited micro-pitted, roughened surfaces, whereas APA-only brackets showed smoother topography with minor residual resin. Despite this improvement, the SBS of HF-treated rebonded brackets remained significantly lower than that of new brackets, in agreement with prior reports that reconditioning protocols cannot fully restore the original bond strength [11, 20].

Previous research has emphasized the importance of combining both mechanical and chemical treatments for more consistent bonding performance. Grosch et al. (2023) [10] reported that dual protocols produce more reliable SBS outcomes than single-method approaches. Similarly, Kim et al. (2024) [12] demonstrated that modifying the bracket base retention design or introducing additional surface-conditioning steps significantly enhances the bonding potential of rebonded ceramic brackets. These findings suggest that the combination of APA and HF conditioning, while not fully restorative, can improve the micromechanical integrity of the bracket base and provide a more predictable bond.

Clinically, the implications of these findings must be interpreted with caution. Although HF conditioning improved rebonding outcomes relative to APA alone, neither approach achieved the SBS of new brackets. Furthermore, the use of HF presents significant handling hazards and limited clinical practicality because of its corrosive and toxic nature [8]. Alternative and safer reconditioning strategies—such as silane-only application, plasma surface treatment, or laser-based methods like Er: YAG have shown promise for achieving clinically acceptable bond strengths while minimizing bracket damage and operator risk [13, 14].

### Failure mode observation

Visual examination under 3.5× magnification revealed that most adhesive remnants remained on the bracket base in both rebonded groups, indicating a cohesive failure within the adhesive layer. This observation supports the SEM pilot findings and aligns with previous studies reporting similar failure behavior in rebonded ceramic brackets [9, 16]. Quantitative ARI scoring was not performed in this study to maintain focus on bracket base conditioning efficiency; however, its inclusion is recommended for future investigations to characterize failure modes more precisely.

### Study limitations

This in vitro study was conducted under controlled laboratory conditions, and thermocycling or long-term water storage were intentionally excluded to isolate the effects of the investigated surface treatments. Although this approach allowed precise control of experimental variables, it limits the extrapolation of the findings to clinical conditions due to the absence of intraoral aging simulation [7]. In addition, only monocrystalline ceramic brackets were evaluated, and enamel curvature variations were not standardized in accordance with DIN 13990 [10]. Furthermore, the adhesive remnant index (ARI) was not assessed, which could have provided additional information on the failure mode.

Another limitation of the present study is that phosphoric acid etching was not performed during the initial bonding of the rebonded groups (Groups B and C), following previously published in vitro protocols. This methodological approach differs from routine clinical bonding procedures and may have influenced the amount and distribution of residual adhesive on the debonded bracket bases. Therefore, the findings should be interpreted within the context of standardized laboratory conditions and immediate shear bond strength evaluation.

### Implications and future directions

Despite these limitations, the findings establish important baseline data for optimizing ceramic bracket recycling. New brackets consistently achieved superior SBS compared with rebonded ones. HF conditioning produced a modest but measurable improvement over APA alone, yet neither method restored the original bond strength. Considering HF’s toxicity and handling risks [8], future research should explore safer and more effective surface treatments such as silane-only application, plasma modification, or laser conditioning (e.g., Er: YAG). Incorporating thermocycling and in vivo testing will also help validate the long-term clinical performance of rebonded brackets.

Overall, this study contributes to a better understanding of how surface-conditioning protocols influence the rebonding performance of ceramic brackets.

## Conclusions

Based on the findings of this in *vitro* study, the following conclusions were drawn:

1. The null hypothesis stating that “there is no significant difference in shear bond strength among the groups” was rejected. Statistically significant differences were observed between new and rebonded monocrystalline ceramic brackets, indicating that surface-conditioning protocols substantially influence bonding outcomes.
2. New ceramic brackets exhibited significantly higher shear bond strength (SBS) than rebonded brackets, confirming their superior adhesive performance under standardized laboratory conditions.
3. Hydrofluoric acid (HF) conditioning produced a moderate improvement in the SBS of rebonded brackets compared with APA alone; however, neither method restored the bond strength to the level of new brackets.
4. Within the limitations of this in vitro design, these findings provide baseline laboratory evidence on the effectiveness of different surface-conditioning methods for rebonding ceramic brackets. Future studies incorporating artificial aging (thermocycling), Adhesive Remnant Index (ARI) analysis, and in vivo validation are necessary to confirm the long-term clinical applicability of these results.

## Recommendations

Based on the findings and within the limitations of this in vitro study, the following recommendations are proposed:

1. Future studies are encouraged to include artificial aging protocols such as thermocycling and long-term water storage to simulate intraoral conditions and assess bond durability over time.
2. In vivo and clinical investigations are recommended to determine whether the observed laboratory improvements translate into reliable clinical performance under functional and environmental stresses.
3. Further research should explore safer and more effective surface-conditioning methods, including plasma treatment, laser etching, and silane-only application, to enhance the shear bond strength (SBS) of rebonded ceramic brackets.
4. Comparative studies should evaluate the effects of different adhesive systems and bracket materials—including polycrystalline ceramic and metallic brackets—to broaden the applicability of these findings.
5. Future investigations should also examine additional parameters such as the Adhesive Remnant Index (ARI), failure modes, and enamel surface alterations following rebonding to provide a more comprehensive understanding of bonding behavior.

## Supplementary Information

Supplementary Material 1.

## Data Availability

The datasets used and analysed during the current study are available from the corresponding author on reasonable request.

## Abbreviations

ANOVA: Analysis of variance
APA: Airborne particle abrasion
ARI: Adhesive remnant index
CEJ: Cementoenamel junction
HF: Hydrofluoric acid
IRB: Institutional review board
kN: Kilonewton
kPa: Kilopascal
MPa: Megapascal
N: Newton
SBS: Shear bond strength
SD: Standard deviation
SEM: Scanning electron microscopy
TCB: Tungsten carbide bur
UTM: Universal testing machine
