# Supplementary material for: Comparison of shear bond strength of new and rebonded ceramic brackets with and without hydrofluoric acid conditioning: an in vitro study
Source: BMC Oral Health. 2026 Feb 7;26:470. doi: 10.1186/s12903-026-07797-7 (PMC12977647; doi:10.1186/s12903-026-07797-7)
Supplement: Supplementary file 1 — Supplementary Material 1. [file 12903_2026_7797_MOESM1_ESM.pdf]

LIST OF APPENDICES

Appendix	Title	Page No.
Appendix A	Teeth are collected and rinsed, and put in Chloramine-T Solution, 0.5%	73
Appendix B	Preparation of the material for the Experiment	74
Appendix C	Metal holder designed to fabricate acrylic blocks	75
Appendix D	Self-Cure Acrylic Resin Preparation, wax knife, Vaseline, and colors	76
Appendix E	Acrylic Mold Preparation and Tooth Embedding with Group Color Coding	77
Appendix F	Monocrystalline Ceramic brackets from AO (AMERICAN ORTHODONTICS).	78
Appendix G	9.5% Hydrofluoric Acid and Primer	79
Appendix H	Micro sandblaster and aluminum oxide powder	80
Appendix I	tungsten carbide bur and low-speed handpiece	81
Appendix J	3M adhesive Transbond XT with adhesive bond	82
Appendix K	37% phosphoric acid gel from Bisco USA	83
Appendix L	Electronic Ruler	84
Appendix M	Sandblaster Box Design with Stand and Bracket Holder	85
Appendix N	Another instrument	86
Appendix O	light cure	87
Appendix P	Groups with their material will be used	88
Appendix Q	Supervisor Overseeing Experimental Procedures in the clinic.	89
Appendix R	Bonding procedure	90
Appendix S	Teeth after bonding	91
Appendix T	Debonding procedure	92
Appendix U	Tungsten carbidebur procedure	93
Appendix V	micro sandblaster procedure	94
Appendix W	Panel Supervision of Experimental Procedure in Clinic.	95

LIST OF APPENDICES

Appendix	Title	Page No.
Appendix X	Hydrofluoric acid application	96
Appendix Y	Incubation of samples	97
Appendix Z	Testing by scanning electronic microscope	98
Appendix AA	Analysis of samples by SEM	99
Appendix BB	Debonding by UTM	103
Appendix CC	Speed used in the UTM	104
Appendix DD	Certificate to use UTM for shear bond strength of rebonded bracket.	105
Appendix EE	Certification Of the Statistician	108
Appendix FF	Present samples to the panel	109
Appendix GG	Panel signature in monitoring the steps of the experiment.	110
Appendix HH	Receipt for SEM	111
Appendix II	Receipt for UTM	112
Appendix JJ	Gantt Chart	113
Appendix KK	Budget Estimation.	114
Appendix LL	Turnitin Similarity Test Results	115
Appendix MM	Certificate of Proofreading	116
Appendix NN	Receipts of material	117
Appendix OO	Curriculum Vitae	121

Appendix A

Teeth are collected, rinsed, and put in Chloramine-T Solution, 0.5%

.

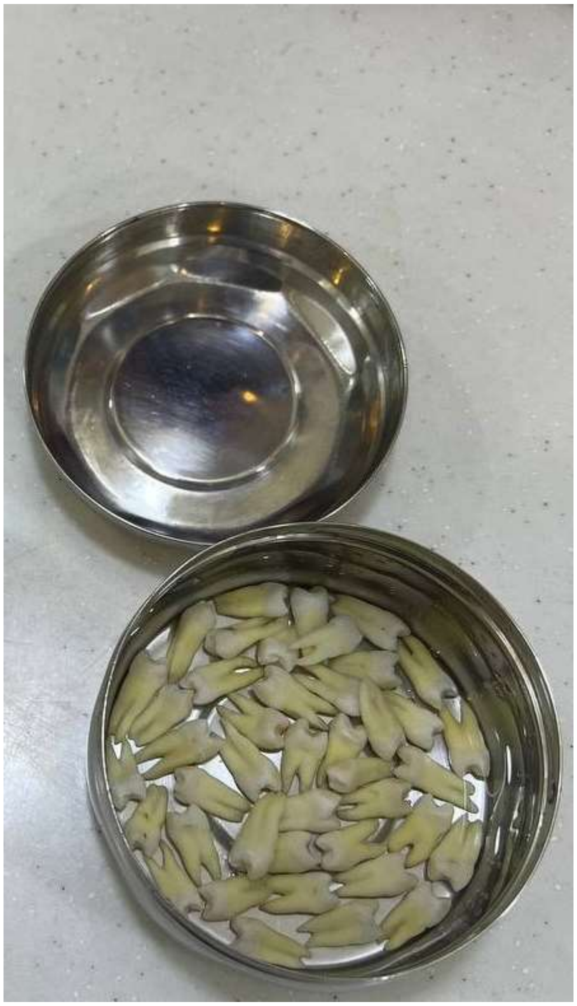

Appendix B

Preparation of the material for the Experiment: Explanation of Material Segmentation and Arrangement

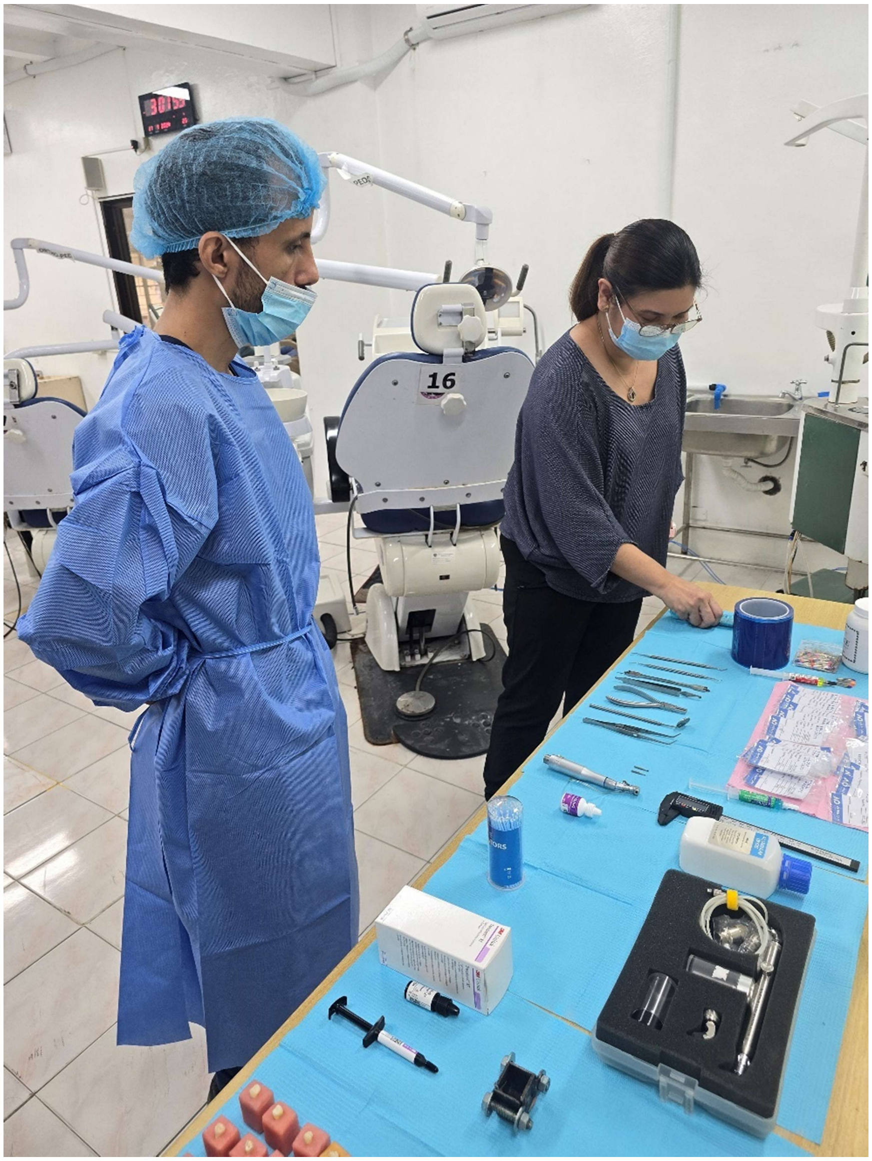

Appendix C

Metal holder designed to fabricate acrylic blocks (25 mm diameter and 30 mm height).

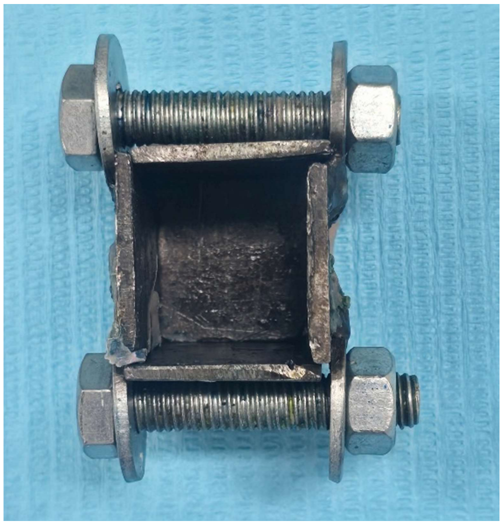

Appendix D

Self-Cure Acrylic Resin Preparation liquid monomer and a powdered polymer.

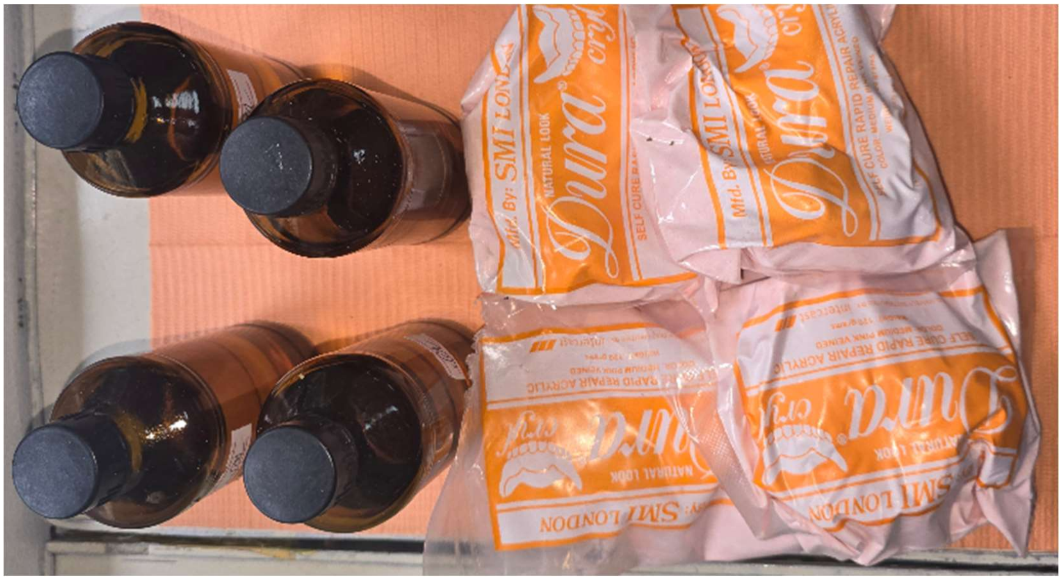

a **wax knife** is being used to mix the self-cure acrylic resin and Vaseline for isolation

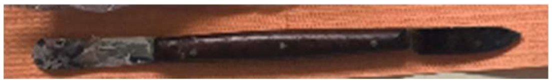

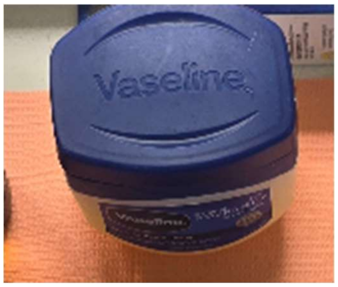

Color Coding for Test Groups (in this image, the test groups are distinguished using color coding for clarity during the experimental process)

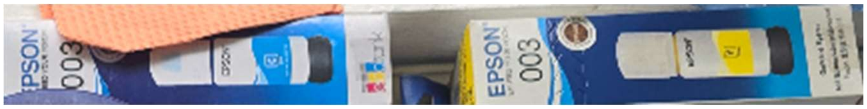

Appendix E

Acrylic Mold Preparation and Tooth Embedding with Group Color Coding: This image illustrates the process of preparing the acrylic molds and color coding the test groups for the experiment. To differentiate between the test groups, each group was assigned a specific color: Group A was marked with pink, Group B with orange.

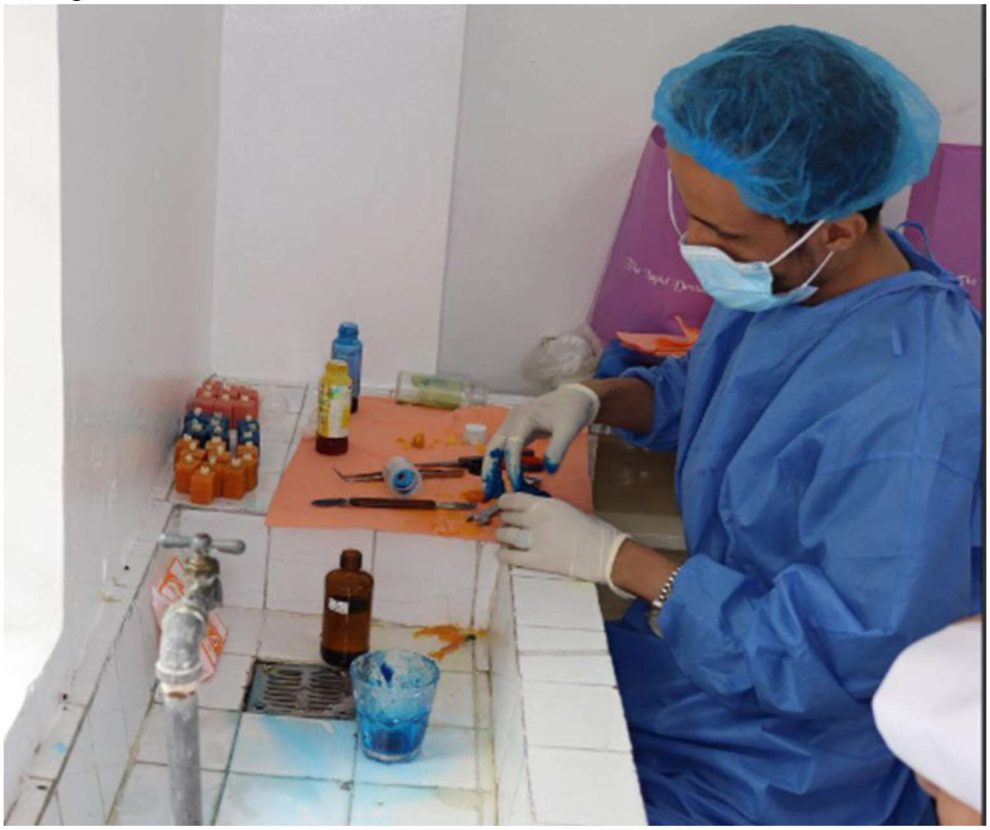

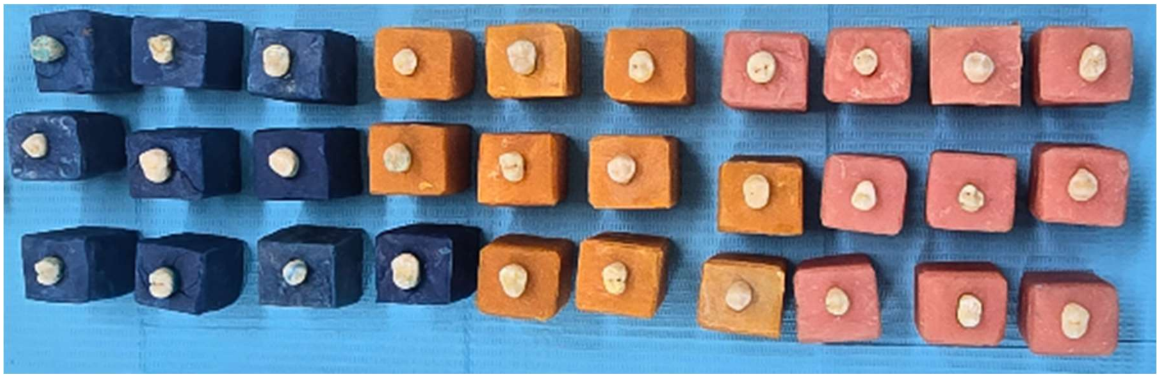

Appendix F

Thirty Monocrystalline Ceramic brackets that have a .022-inch slot purchased from AO (AMERICAN ORTHODONTICS) will be utilized.

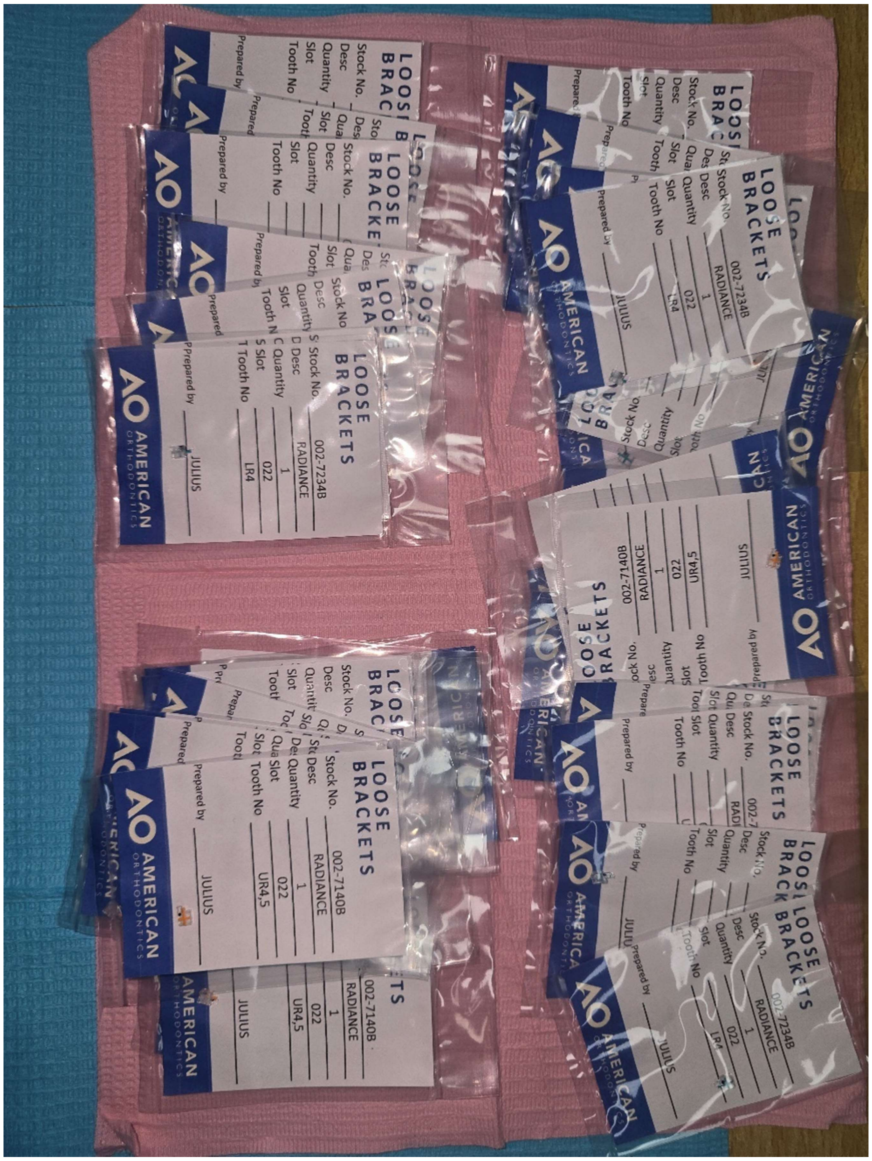

Appendix G

9.5% Hydrofluoric Acid and Primer from Bisco, USA

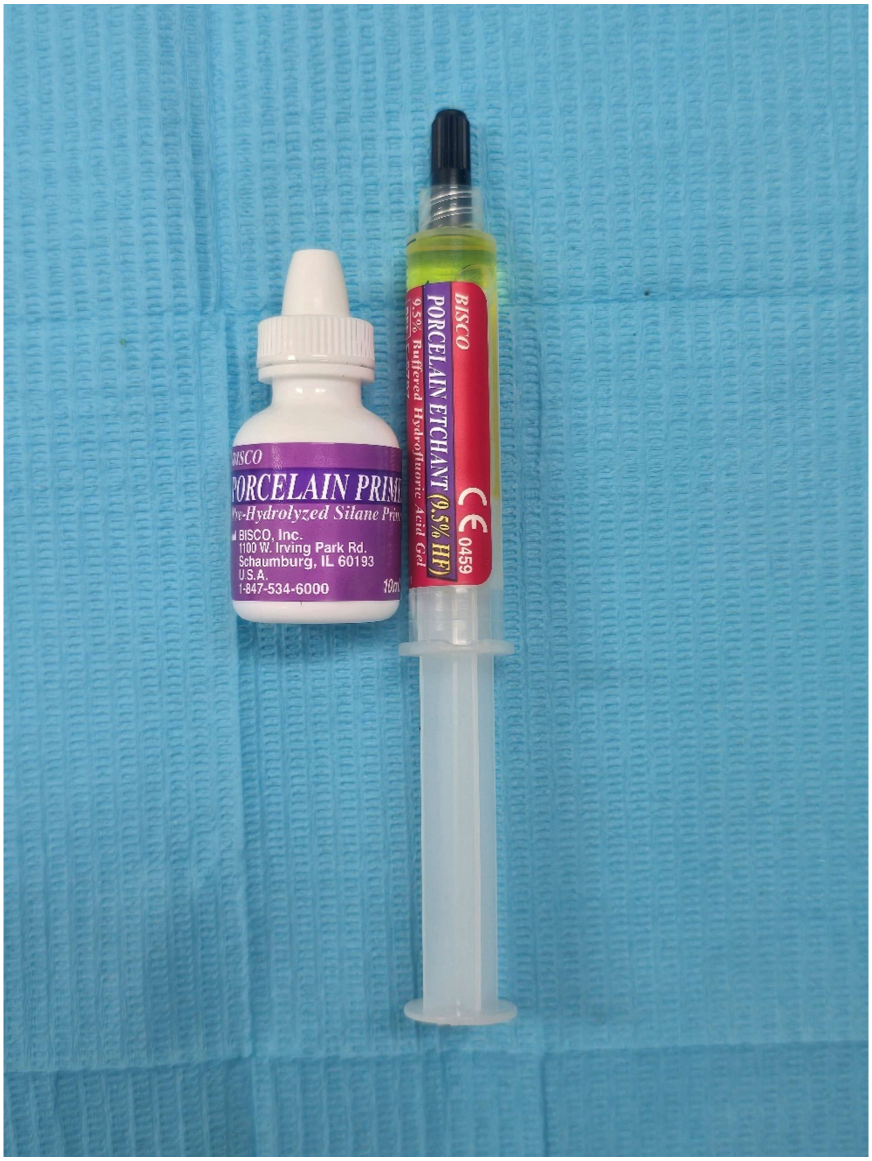

Appendix H

Micro sandblaster and aluminum oxide powder 50micron.

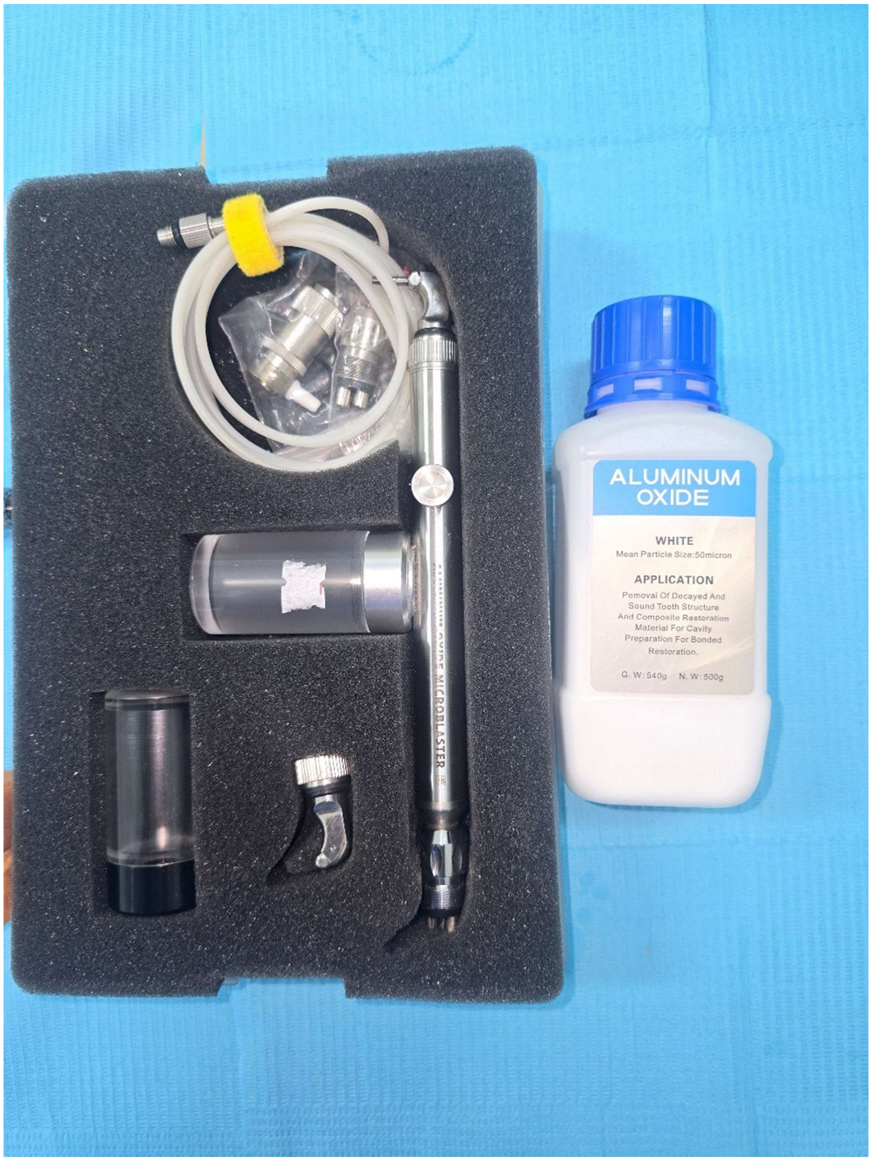

Appendix I

tungsten carbide bur and low-speed handpiece

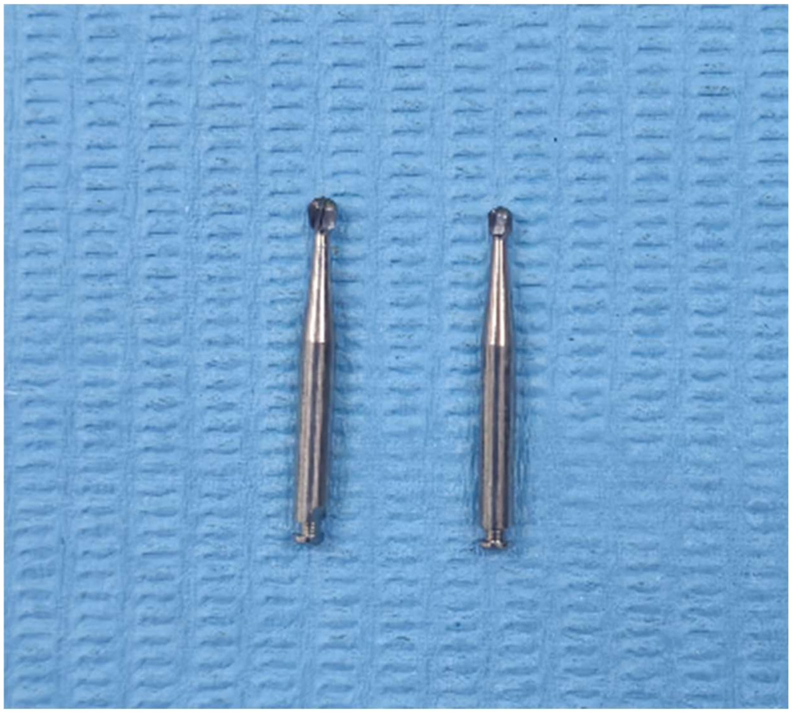

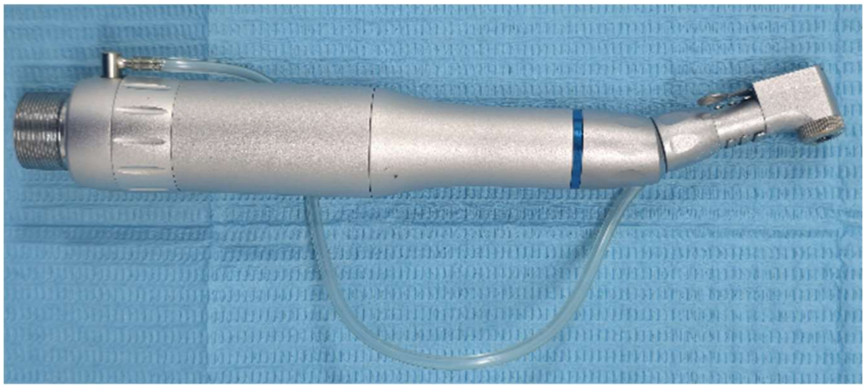

Appendix J

3M adhesive Transbond XT with adhesive bond

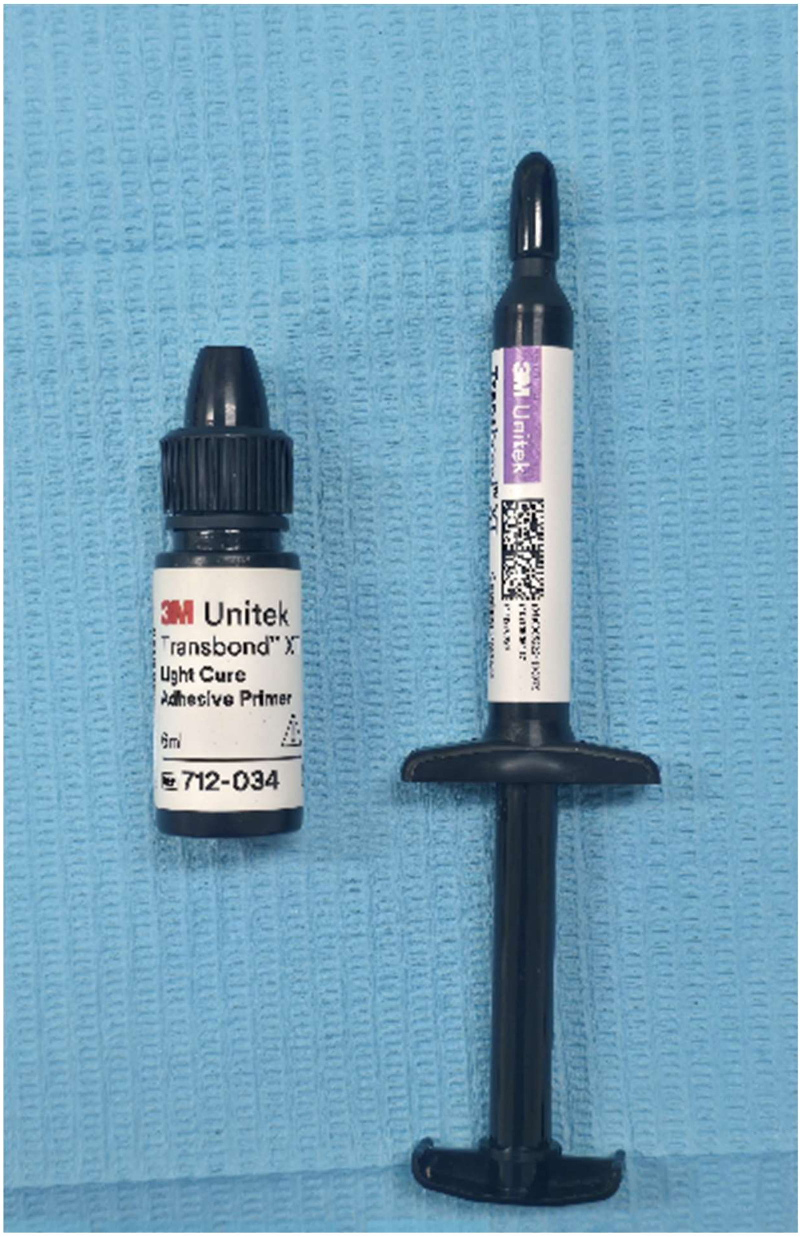

Appendix K

37% phosphoric acid gel from Bisco USA

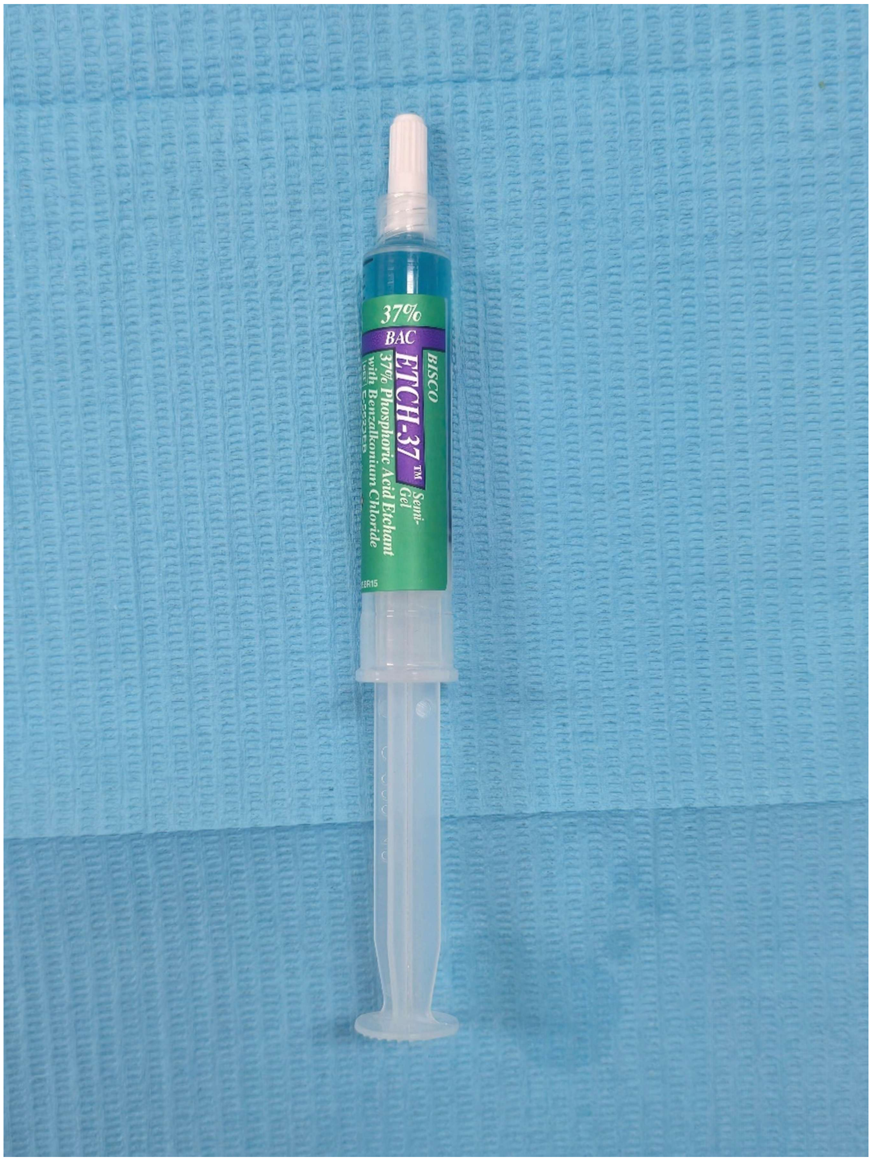

Appendix L

Electronic Ruler for Measuring the Distance Between the Bracket Surface and the Sandblaster

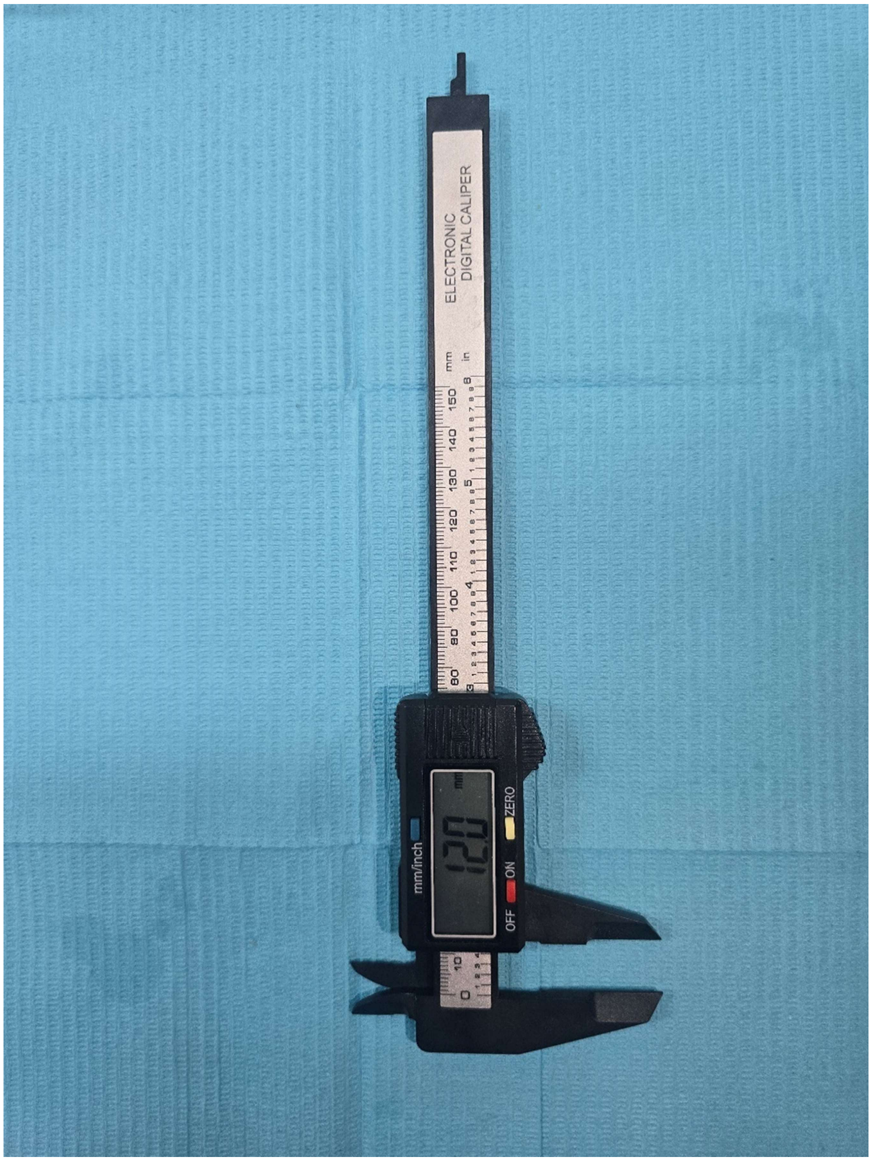

Appendix M

Sandblaster Box Design with Stand and Bracket Holder

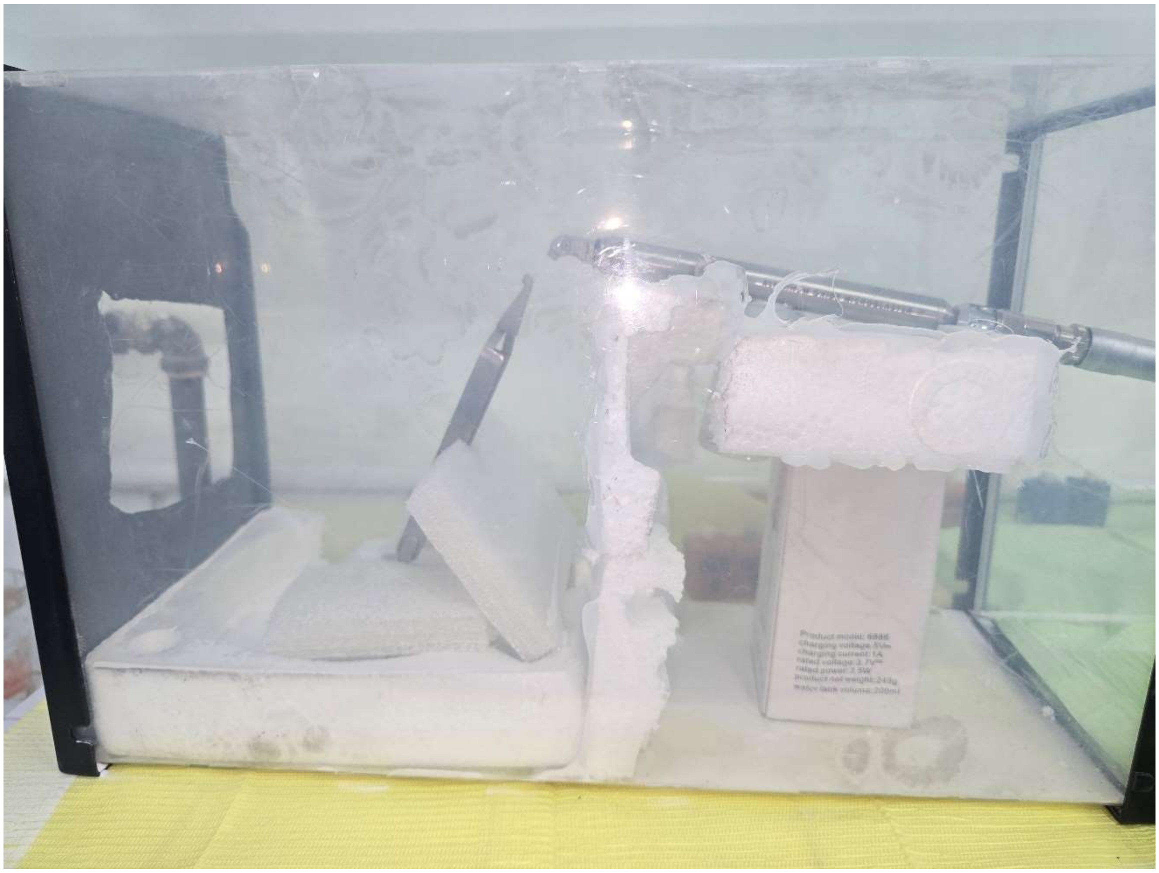

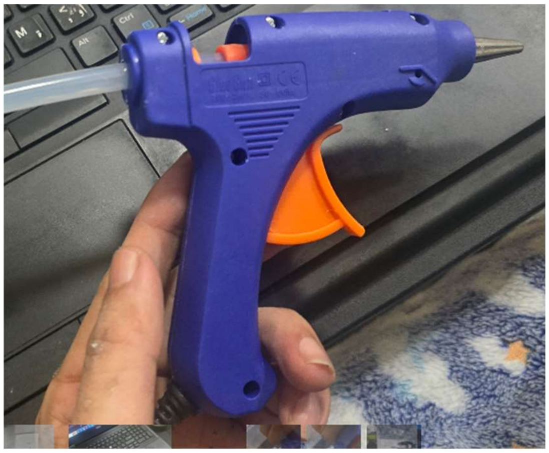

Appendix N

Another instrument

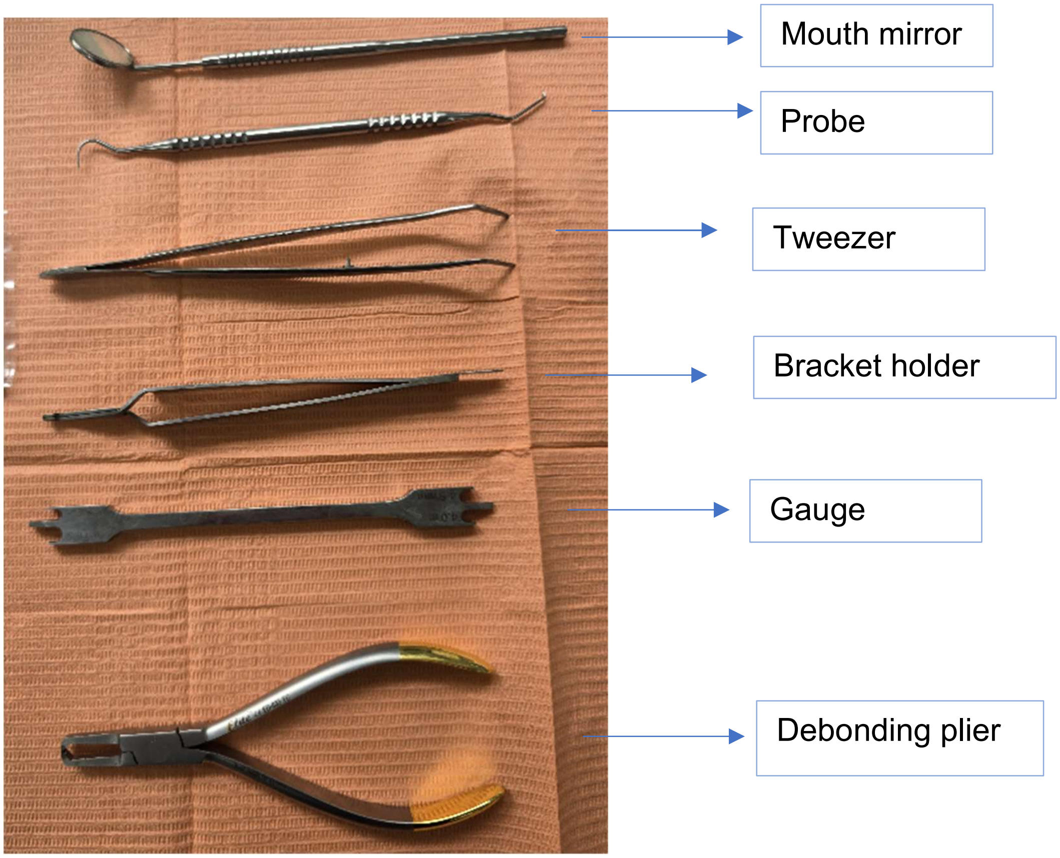

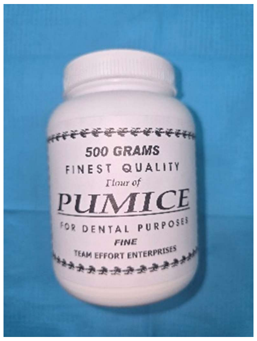

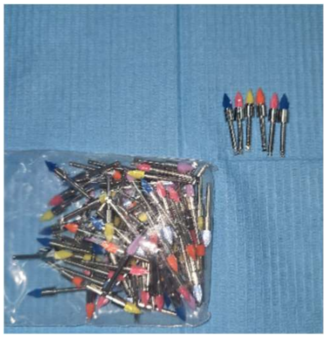

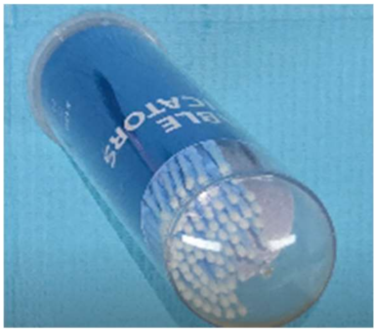

Appendix O

light cure

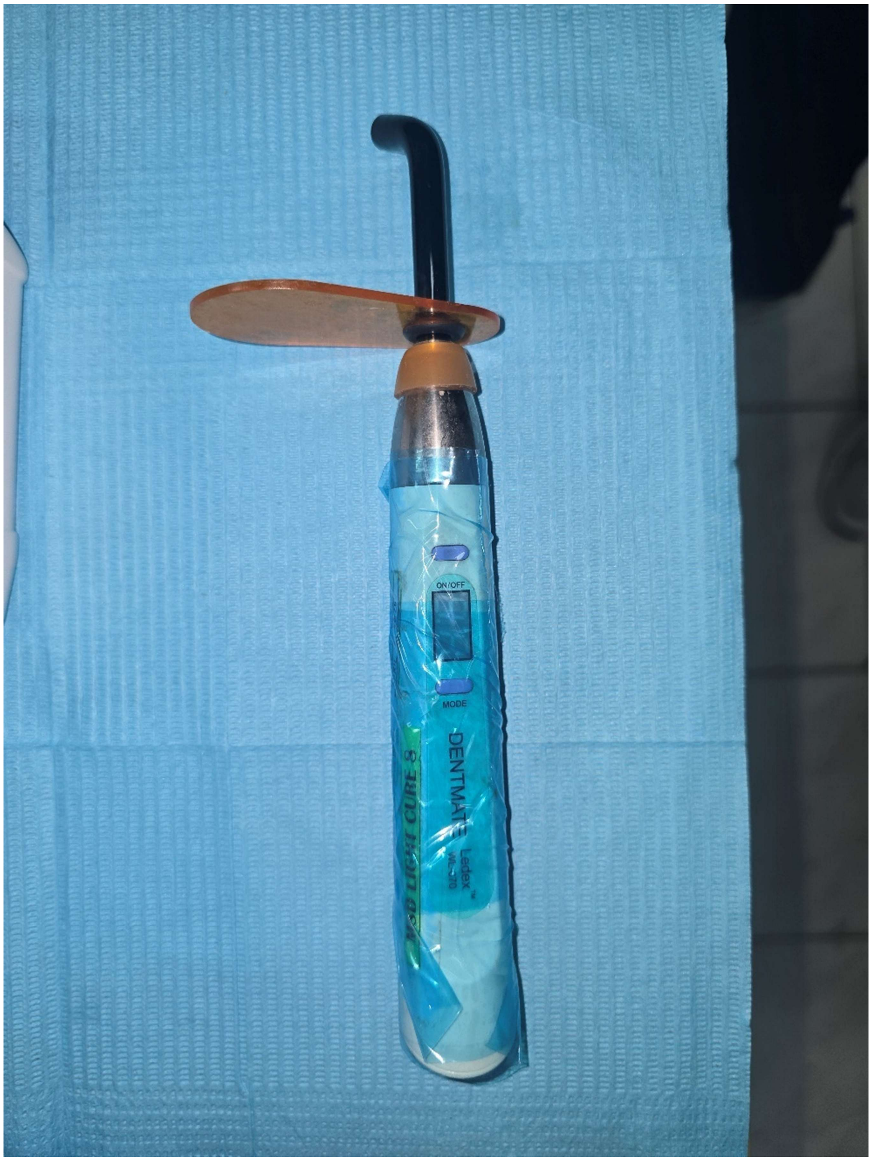

Appendix P

Groups with their material will be used

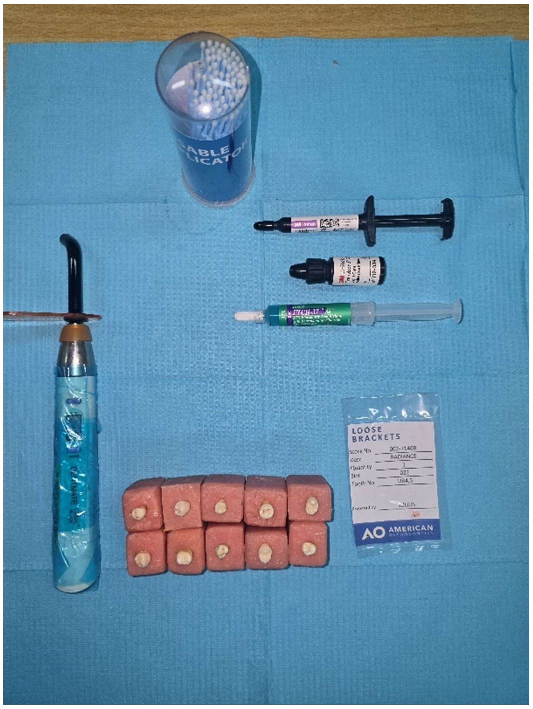

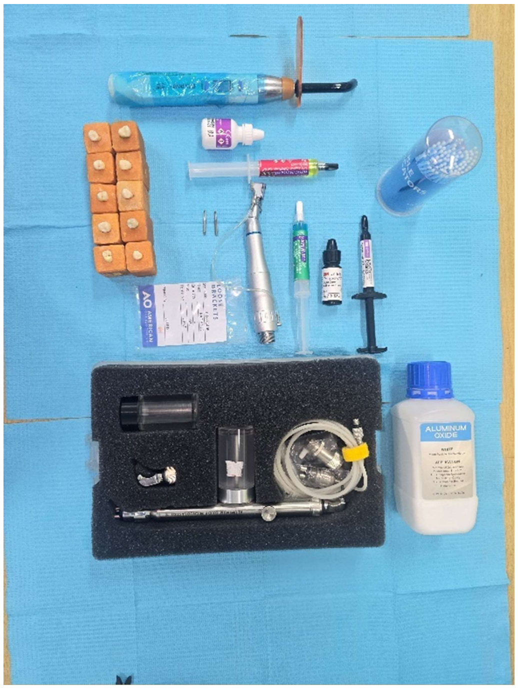

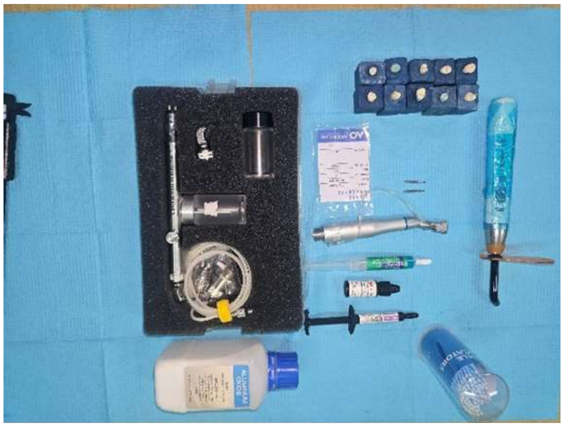

Appendix Q

Supervisor Overseeing Experimental Procedures in the clinic.

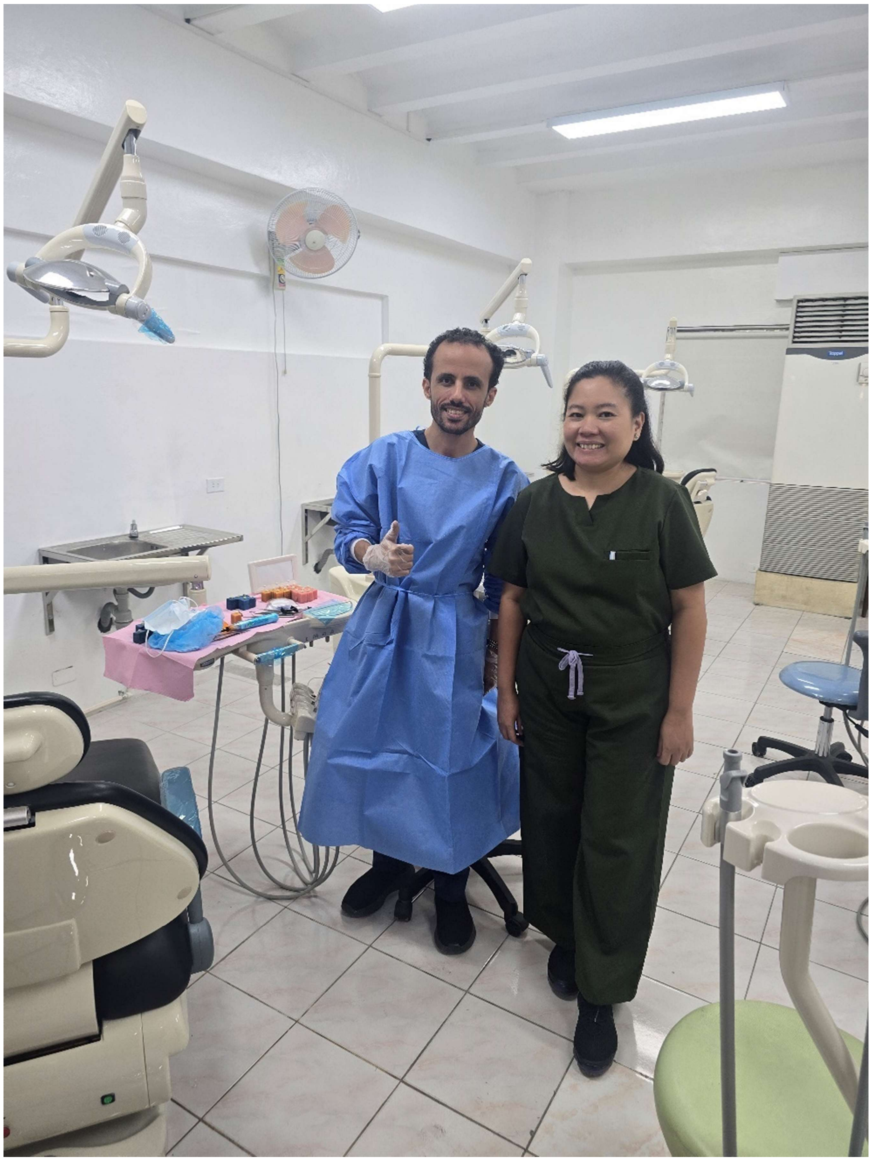

Appendix R

Bonding procedure

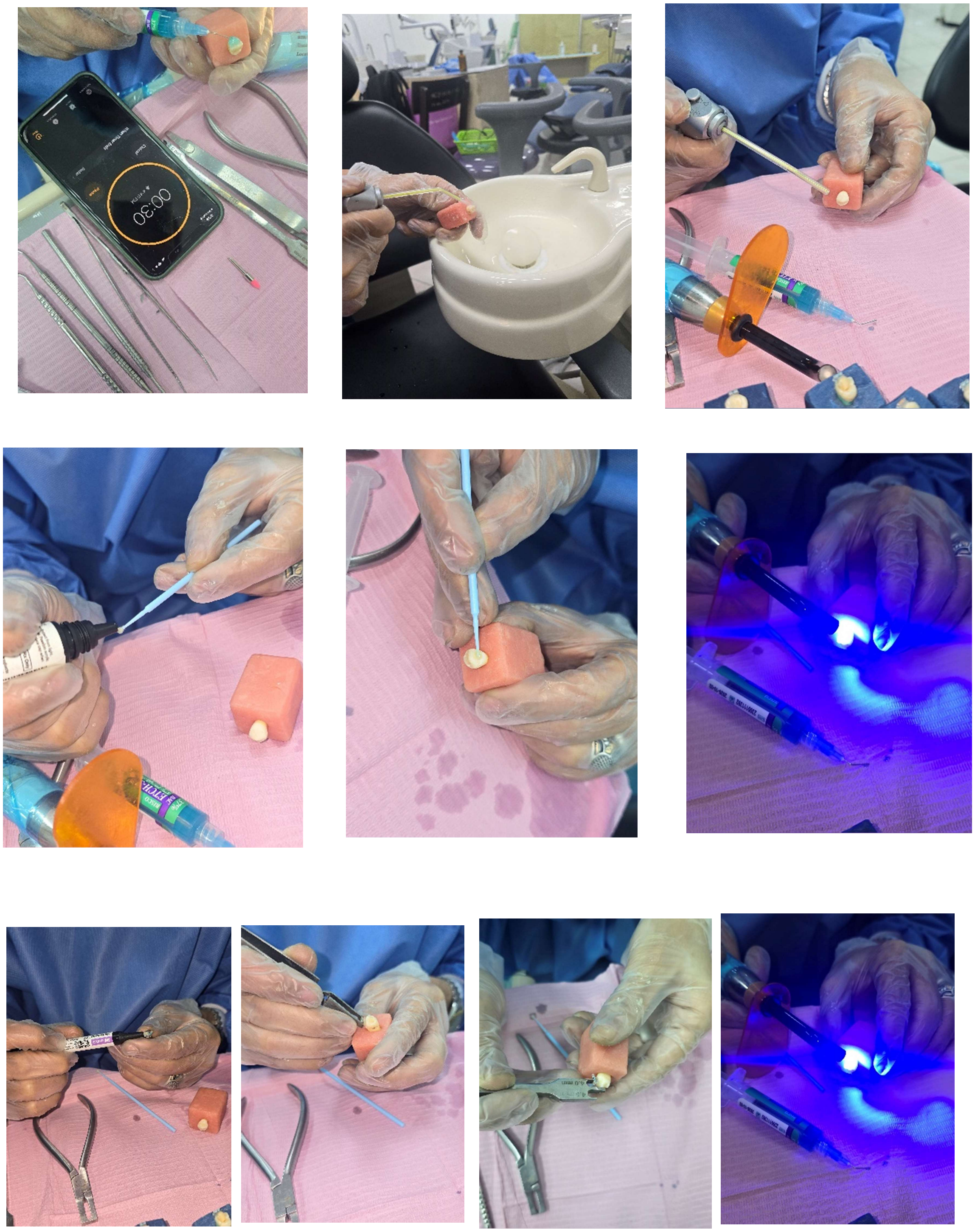

Appendix S

Teeth after bonding

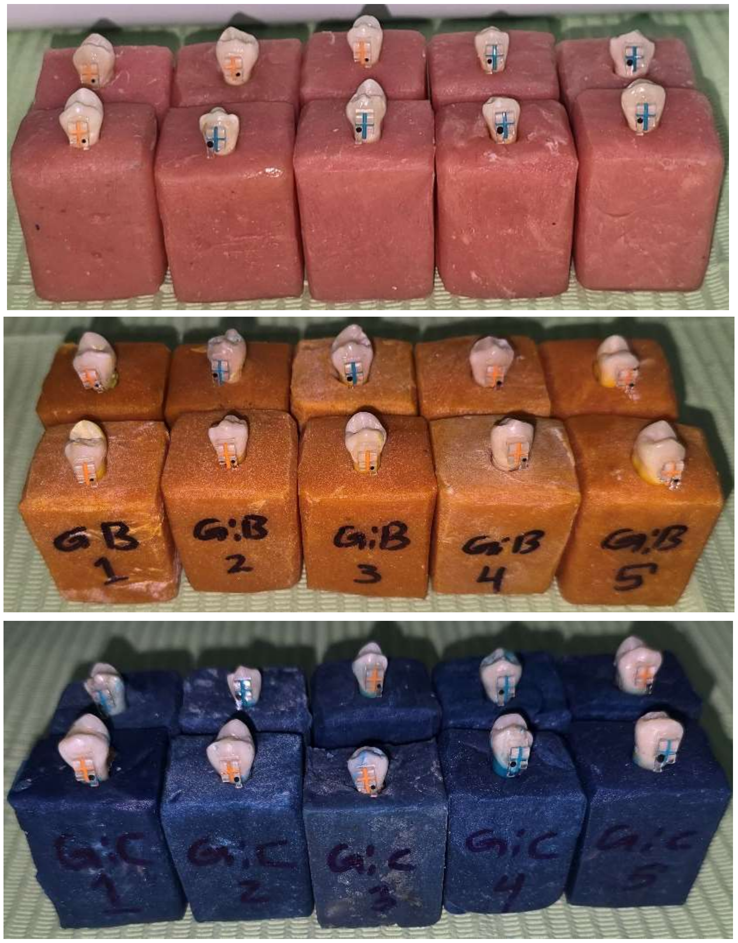

Appendix T

The debonding procedure for the test group was performed by squeezing it with a debonding plier.

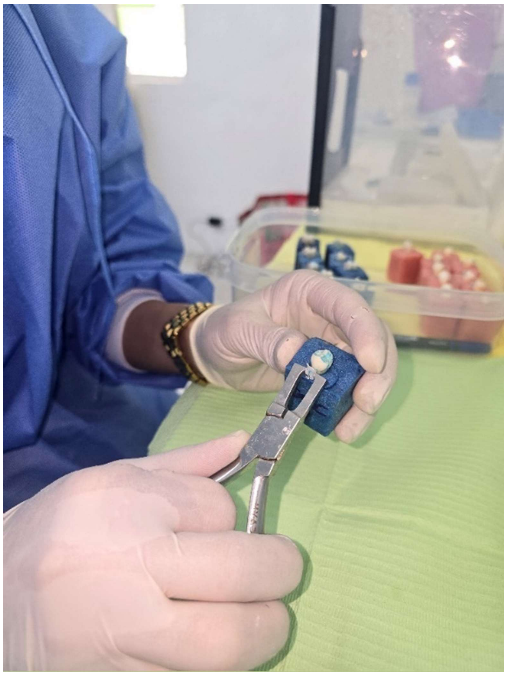

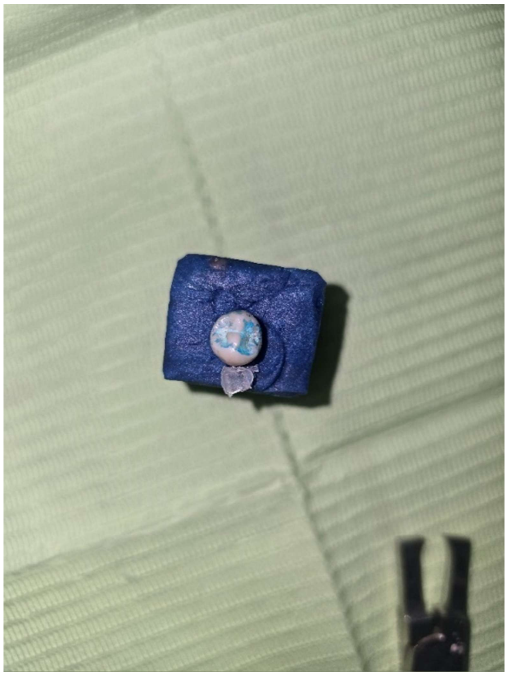

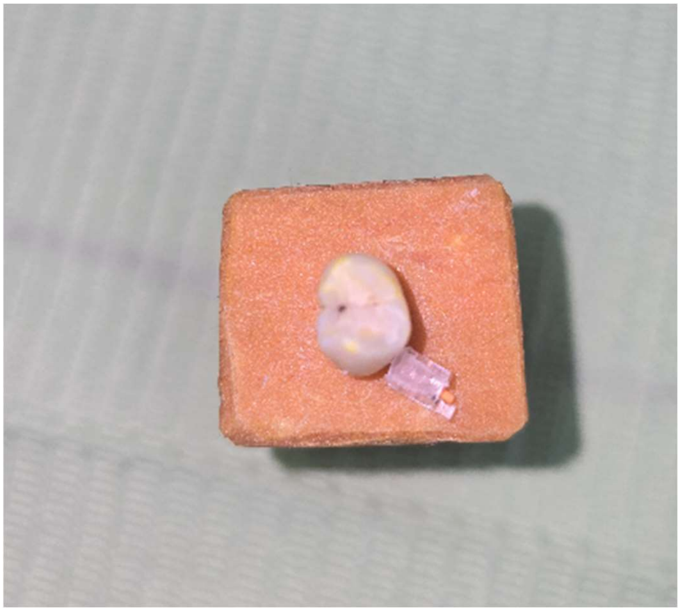

Appendix U

Tungsten carbide procedure

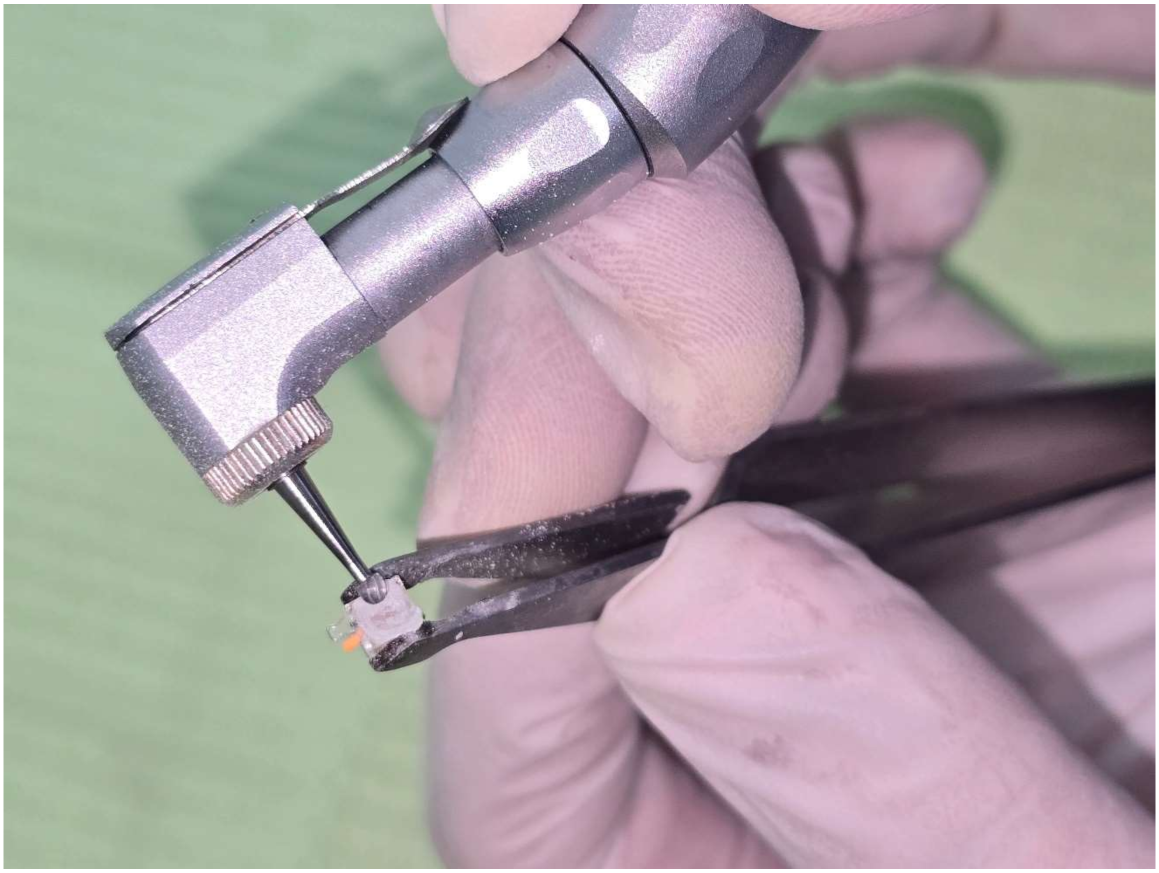

Appendix V

Micro Sandblaster Procedure

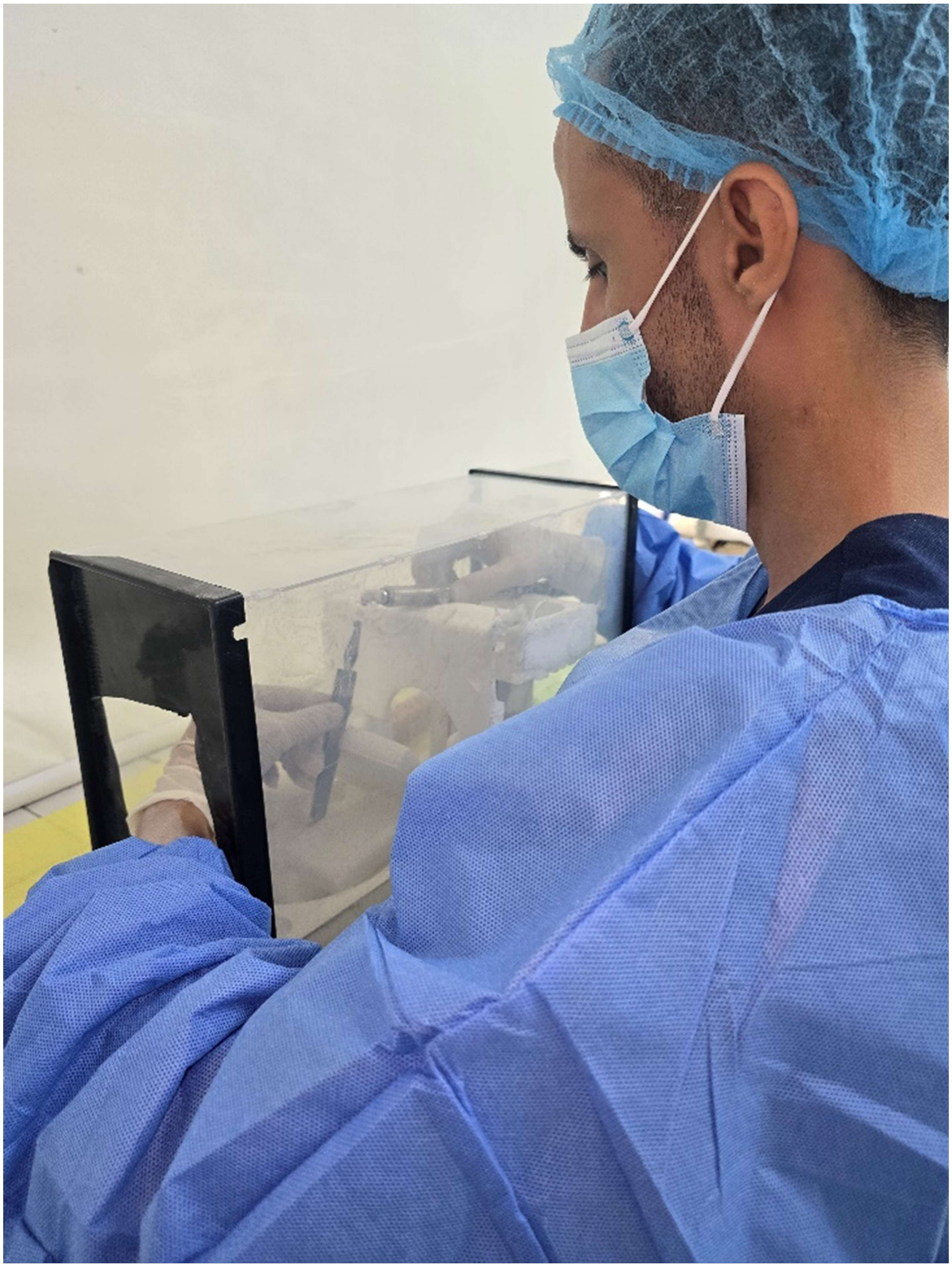

Appendix W

Panel Supervision of Experimental Procedure in Clinic.

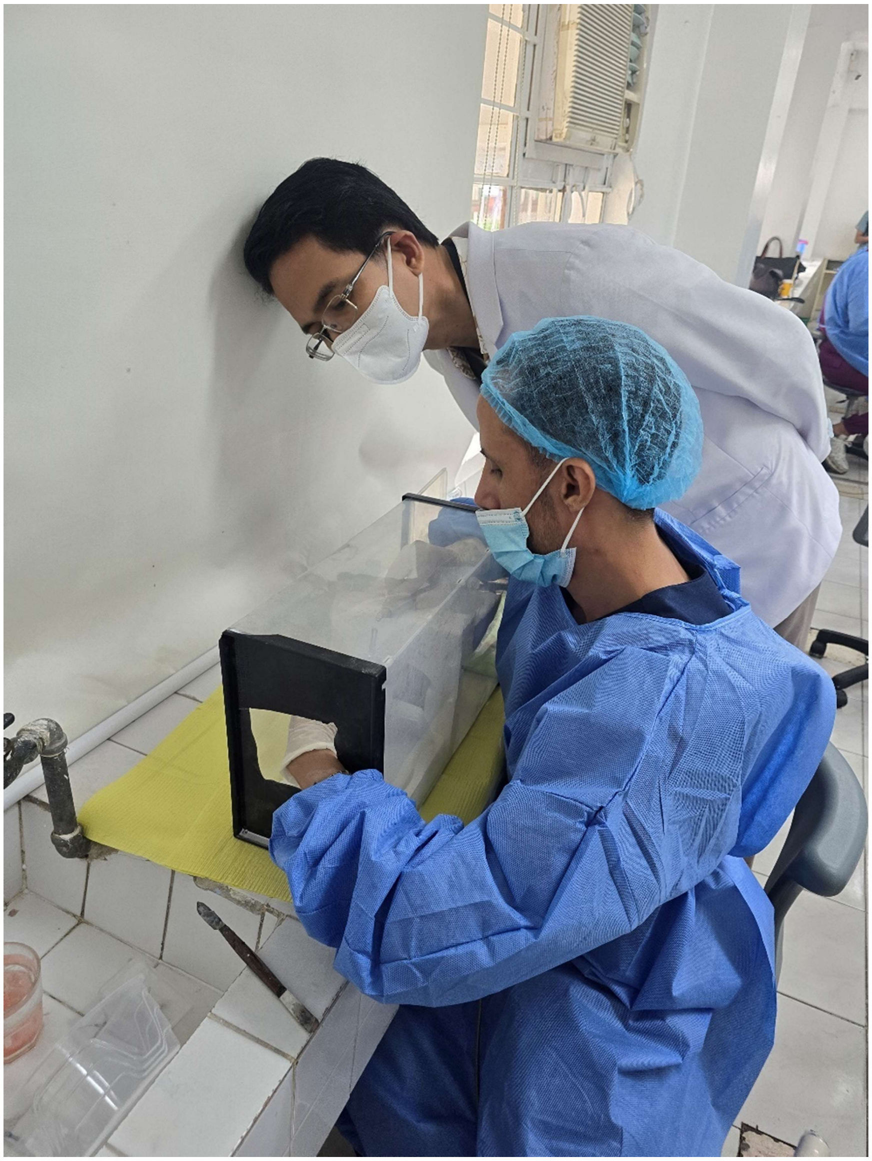

Appendix X

Hydrofluoric acid application

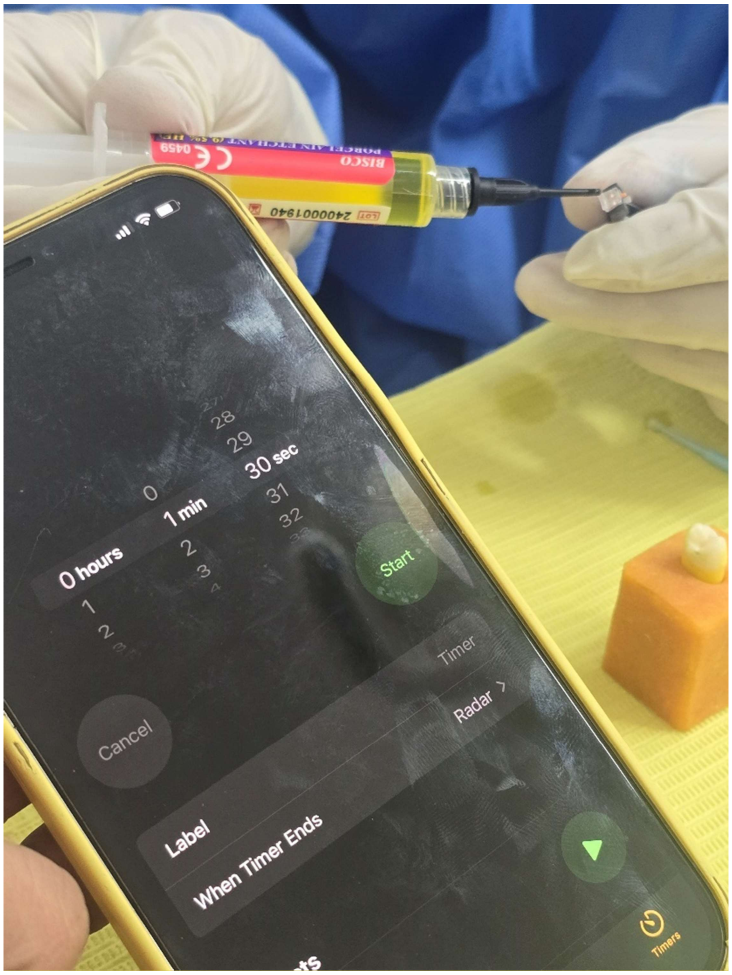

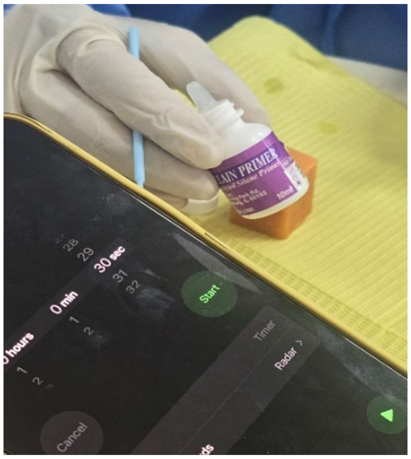

Appendix Y

Incubation of samples

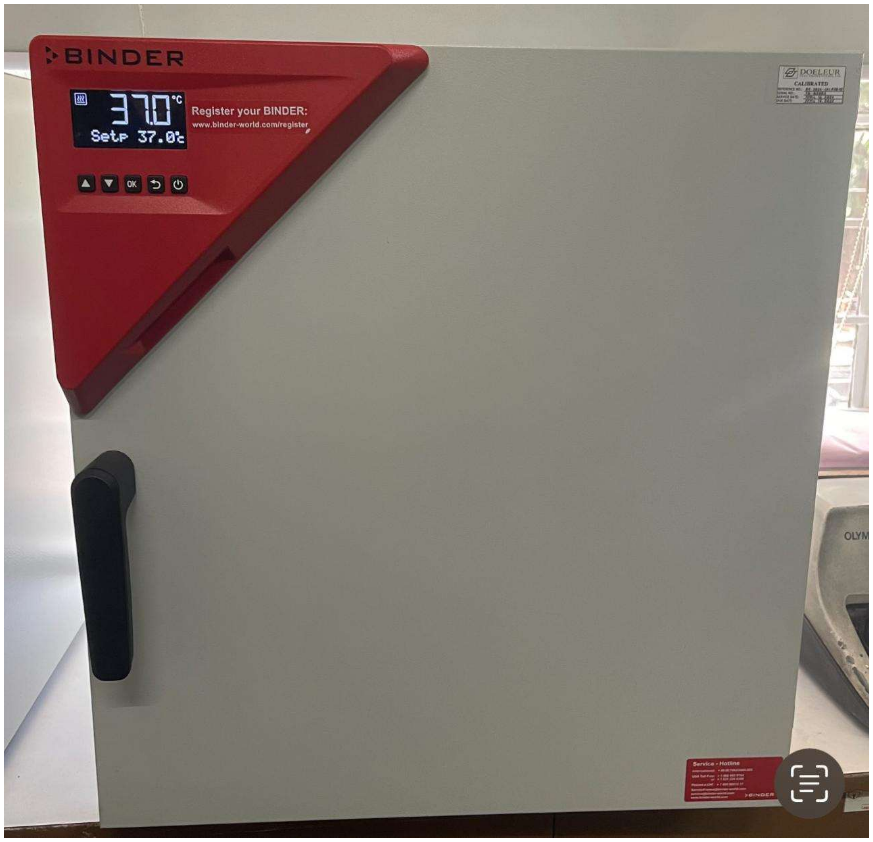

Appendix Z

Testing by scanning electronic microscope

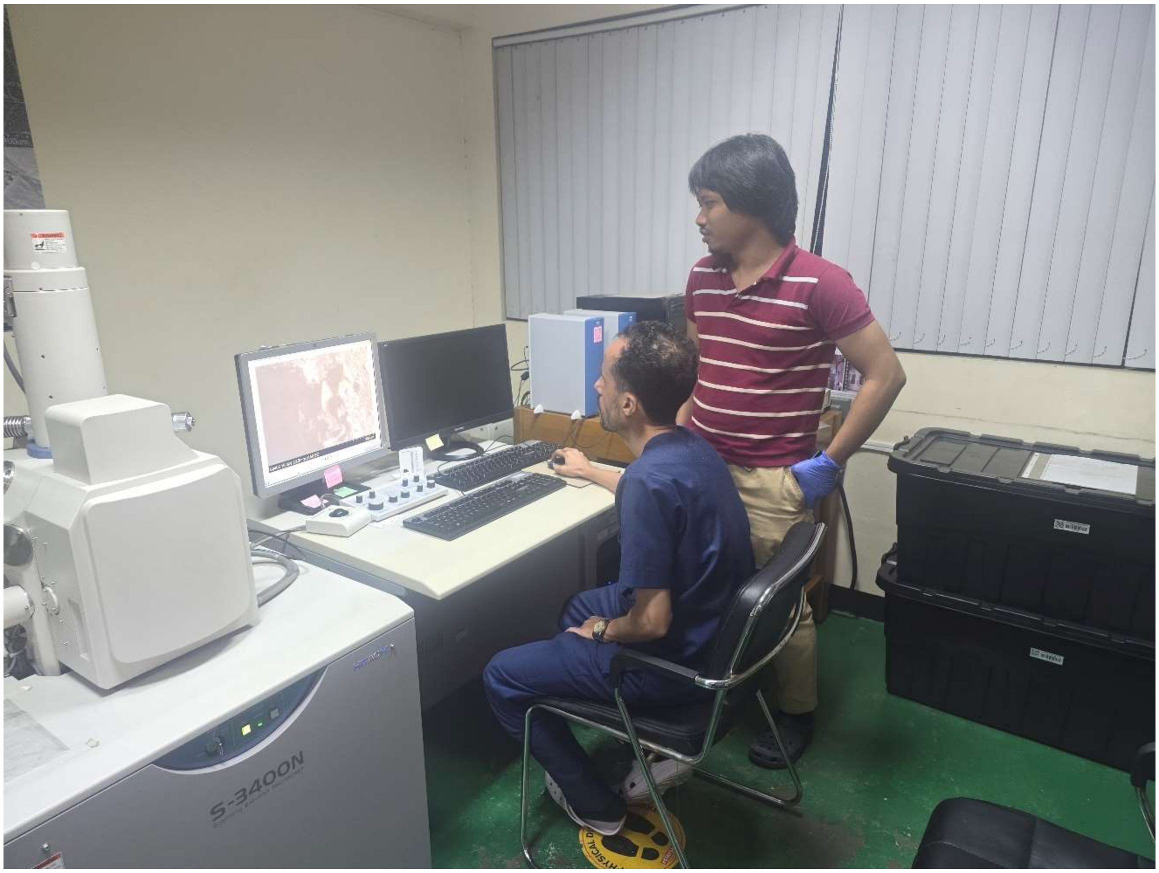

Appendix AA

The samples were analyzed using a Scanning Electron Microscope (SEM) at two magnifications, 500x and 1000x, for a total of 6 samples, with 3 samples from each group.

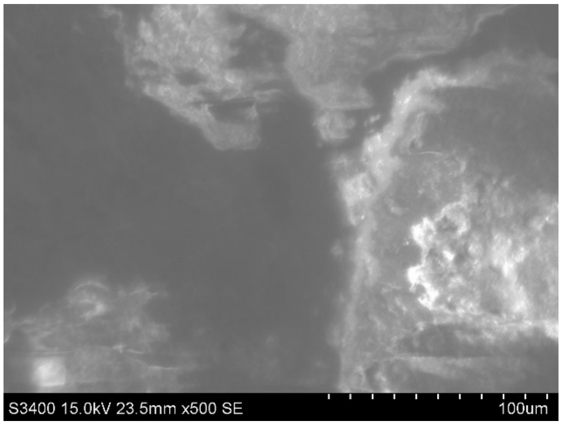

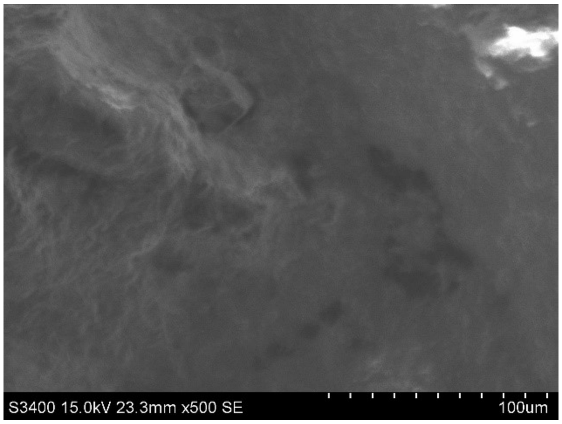

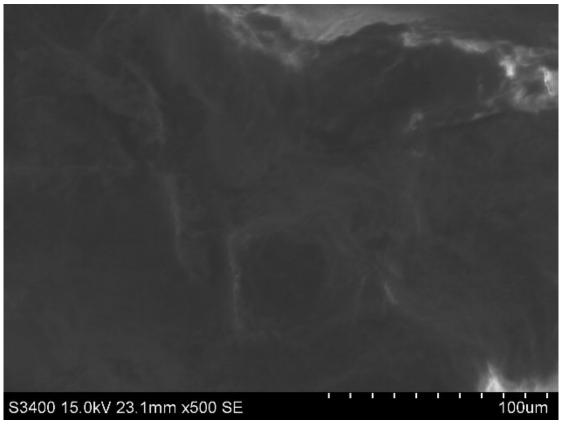

Analysis of sample By SEM for Group B Samples in 500x

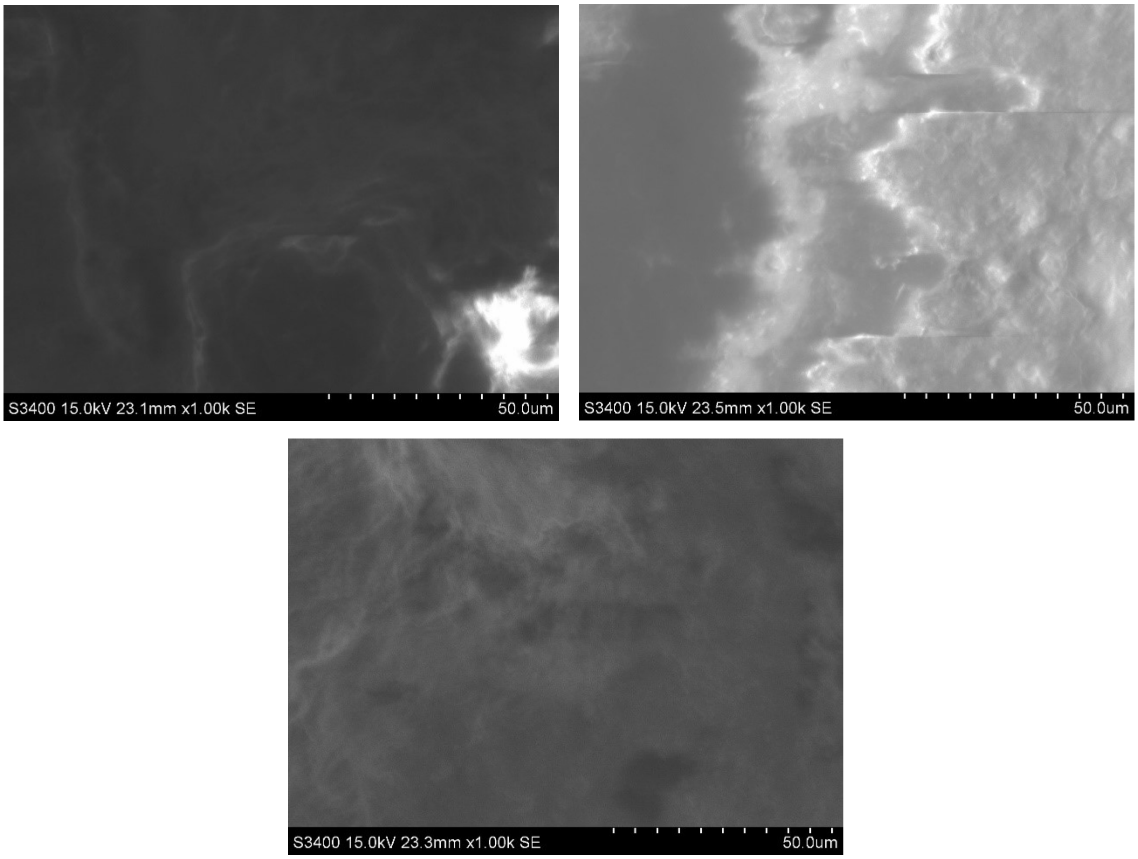

Analysis of sample By SEM for Group B Samples in 1000x

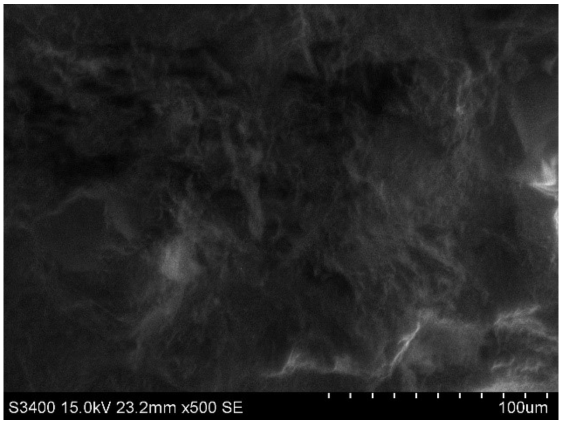

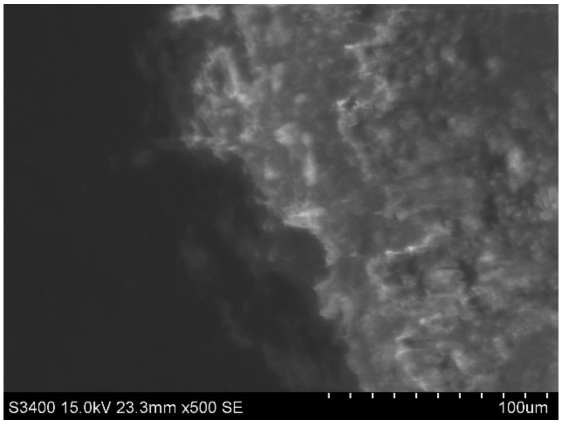

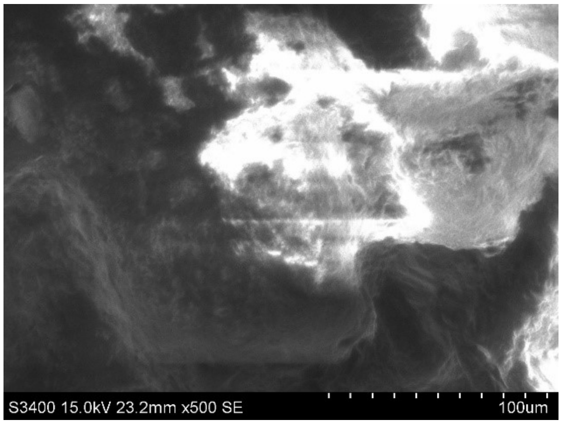

Analysis of sample By SEM for Group C Samples in 500x

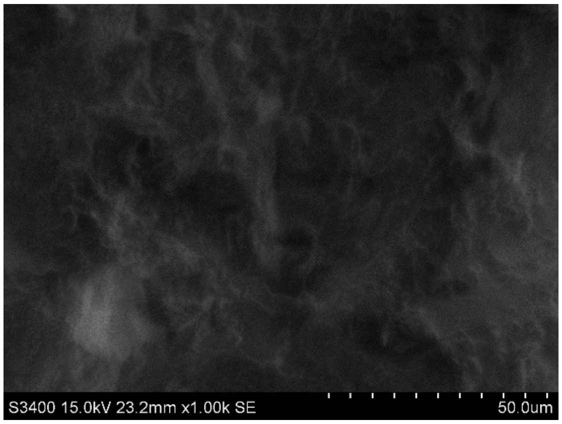

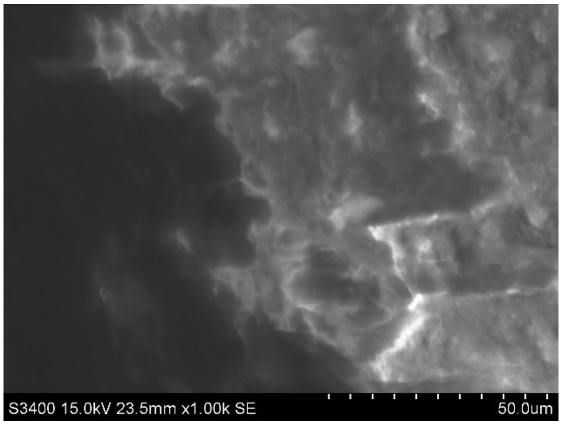

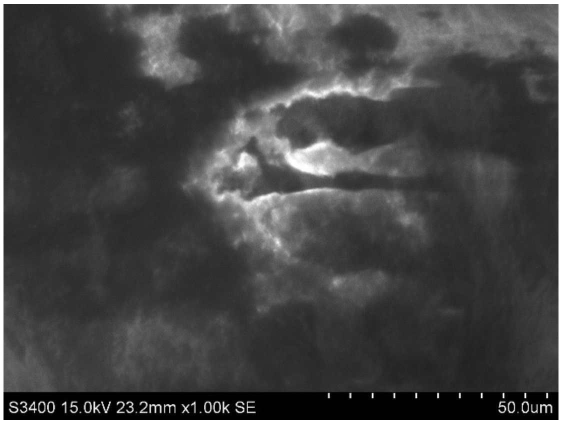

Analysis of sample By SEM for Group C Samples in 1000x

Appendix BB

Debonding was performed using a universal testing machine (INSTRON 100 KN).

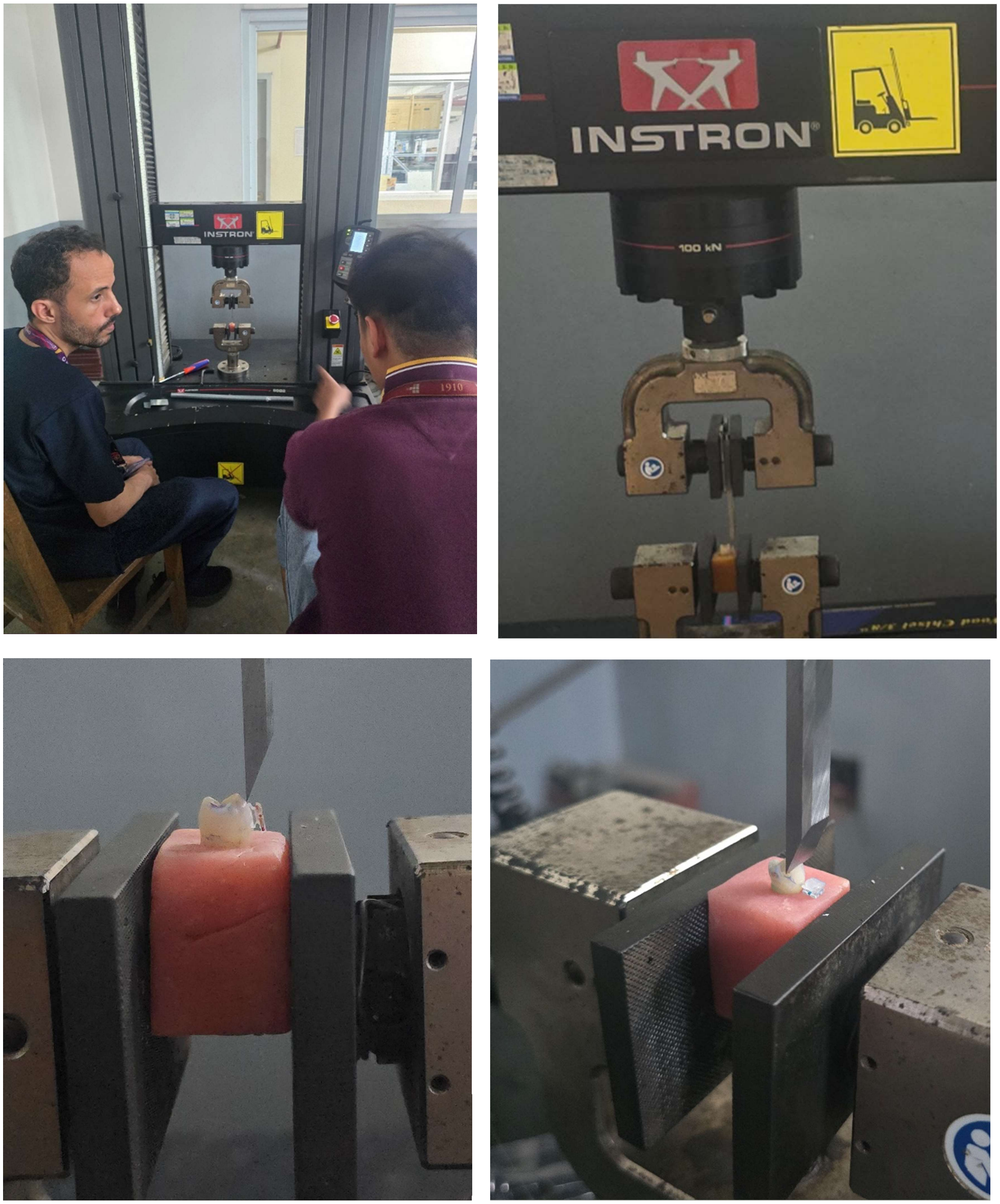

Appendix CC

Speed used in the Universal Testing Machine (UTM) was 0.500 mm/min.

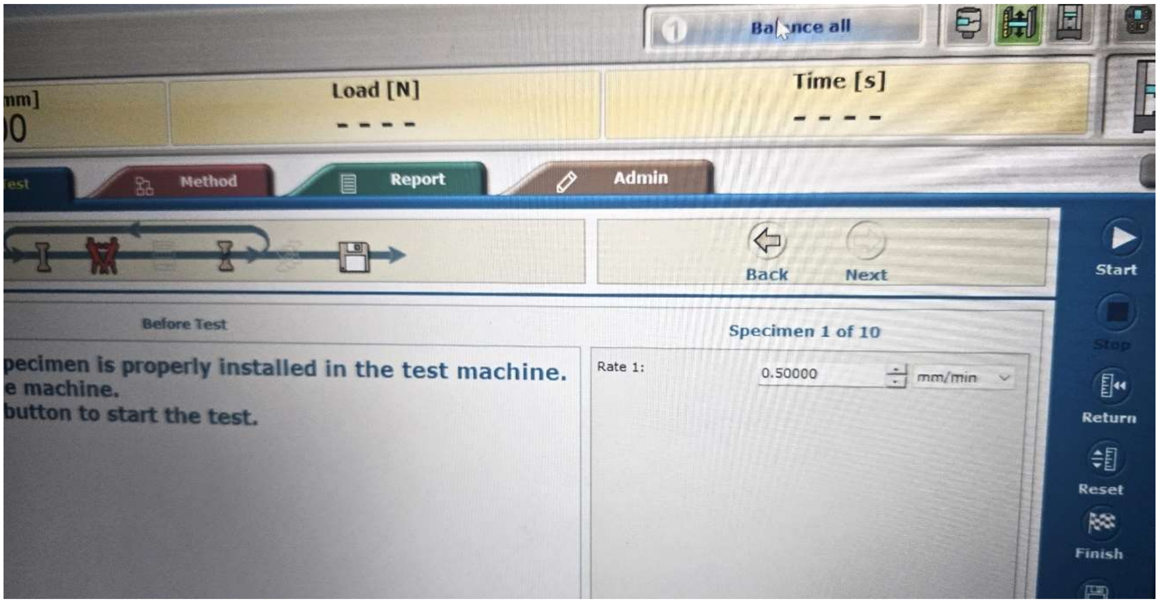

The Universal Testing Machine (UTM) was set to a speed of 0.500 mm/min to apply the load consistently during the shear bond strength testing.

Appendix DD

Certificate to use UTM for shear bond strength of rebonded bracket.

UNIVERSITY OF THE PHILIPPINES DILIMAN
INSTITUTE OF CIVIL ENGINEERING
Construction Materials and Structures Laboratory

REPORT ON ADHESION TEST

Report No. : MCU30 100824
Page No. : 1 of 3
Date : Oct 8, 2024

CLIENT : Alkamel Aqlan Ali Moqbelqaid - MCU

PROJECT : Thesis Project

SAMPLE : Ceramic bracket (Monocrystalline) Group A (pink)

TEST METHOD : Adhesion Test

Sample Identification	Maximum Load (N)
A – 1	329
A – 2	37
A – 3	219
A – 4	262
A – 5	65
A – 6	319
A – 7	157
A – 8	168
A – 9	214
A – 10	141

Note:
1. Speed of testing used is 0.5mm/min.
2. Results provided are specific to the samples tested and obtained following specified methods and standards.
3. Certain results may have been updated following a thorough review and verification of the raw data.
4. It is the responsibility of the client to exercise due diligence in interpreting and utilizing the test results provided.
5. This certificate should not be reproduced, except in full, without the written consent of COMSLAB.

Witnessed By: Alkamel Aqlan Ali Moqbelqaid

Tested By: Mervin Sabdani

Prepared By:
Engr. Robin Cristopher Q. Nabong
Senior Research Associate

Certified Correct By:
Nathaniel B. Diola, Dr. Engg.
Faculty-in-Charge

UP COMSLAB

Construction Engineering Laboratory Building, Institute of Civil Engineering, Pardo de Tavera St.,
University of the Philippines Diliman, Quezon City 1101 NCR, Philippines
Tel. No: +632 981-8500 loc. 3182 E-mail: comslab.upd@up.edu.ph Website: ice.upd.edu.ph

UNIVERSITY OF THE PHILIPPINES DILIMAN
INSTITUTE OF CIVIL ENGINEERING
Construction Materials and Structures Laboratory

REPORT ON ADHESION TEST

Report No. : MCU30 100824
Page No. : 2 of 3
Date : Oct 8, 2024

CLIENT : Alkamel Aqlan Ali Moqbelqaid - MCU

PROJECT : Thesis Project

SAMPLE : Ceramic bracket (Monocrystalline) Group B (orange)

TEST METHOD : Adhesion Test

Sample Identification	Maximum Load (N)
B – 1	97
B – 2	84
B – 3	123
B – 4	52
B – 5	171
B – 6	28
B – 7	146
B – 8	52
B – 9	80
B – 10	89

Note:
1. Speed of testing used is 0.5mm/min.
2. Results provided are specific to the samples tested and obtained following specified methods and standards.
3. Certain results may have been updated following a thorough review and verification of the raw data.
4. It is the responsibility of the client to exercise due diligence in interpreting and utilizing the test results provided.
5. This certificate should not be reproduced, except in full, without the written consent of COMSLAB.

Witnessed By: Alkamel Aqlan Ali Moqbelqaid

Tested By: Mervin Sabdani

Prepared By:

Engr. Robin Cristopher Q. Nabong
Senior Research Associate

Certified Correct By:

Nathaniel B. Diola, Dr. Engg.
Faculty-in-Charge

UP COMSLAB

UNIVERSITY OF THE PHILIPPINES DILIMAN
INSTITUTE OF CIVIL ENGINEERING
Construction Materials and Structures Laboratory

REPORT ON ADHESION TEST

Report No. : MCU30 100824
Page No. : 3 of 3
Date : Oct 8, 2024

CLIENT : Alkamel Aqlan Ali Moqbelqaid - MCU

PROJECT : Thesis Project

SAMPLE : Ceramic bracket (Monocrystalline) Group C (blue)

TEST METHOD : Adhesion Test

Sample Identification	Maximum Load (N)
C – 1	138
C – 2	51
C – 3	29
C – 4	58
C – 5	45
C – 6	91
C – 7	228
C – 8	134
C – 9	79
C – 10	92

Note:
1. Speed of testing used is 0.5mm/min.
2. Results provided are specific to the samples tested and obtained following specified methods and standards.
3. Certain results may have been updated following a thorough review and verification of the raw data.
4. It is the responsibility of the client to exercise due diligence in interpreting and utilizing the test results provided.
5. This certificate should not be reproduced, except in full, without the written consent of COMSLAB.

Witnessed By: Alkamel Aqlan Ali Moqbelqaid

Tested By: Mervin Sabdani

Prepared By:
Engr. Robin Cristopher Q. Nabong
Senior Research Associate

Certified Correct By:
Nathaniel B. Diola, Dr. Engg.
Faculty-in-Charge

Appendix EE

Certification Of the Statistician

Certification of Statistical Treatment

October 9, 2024

I, the undersigned hereby certify that the research paper of **Dr. Aqlan Ali Moqbel Qaid Al-Kamel**, a student of the Graduate School, Manila Central University with the thesis entitled "**Comparison of Shear Bond Strength of New and Re-bonded Ceramic Brackets With and Without Hydrofluoric Acid Conditioning**" was duly computed statistically and scientifically analyzed, using various statistical methods specifically appropriate for the research analysis needed for the study.

Ramil V. Flores Ph.D.

Statistician

Appendix FF

Present samples to the panel

MCU
Manila Central University

MANILA CENTRAL UNIVERSITY
COD/MSD ORTHODONTICS
CALOOCAN CITY

DATE: September - 16- 2024

Sir/ Ma'am,

This is to certify that I presented my sample/ samples to my panel members before the actual experimentation. My thesis is titled **"COMPARISON OF SHEAR BOND STRENGTH OF NEW AND REBONDED CERAMIC BRACKETS WITH AND WITHOUT HYDROFLUORIC ACID CONDITIONING".** which requires thirty samples to be tested in a Universal Testing machine . Thank you.

Respectfully yours,
Dr. Al-kamel Aqlan Ali Modbel Qaid
(Printed name and Signature)

Noted:

Dr. Shirchie Iris P. Galvan
Thesis Adviser

Panel Members:

1. Galahad P. Perea
2.

Appendix GG

Panel signature in monitoring the steps of the experiment.

MCU
Manila Central University

NAME OF RESIDENT: AL-KAMEL AQLAN ALI MOQBEL QAID

DAY:	Time Started:	Time Finish:	Signature of Resident	Name & Signature of CI
18-09-2024	1:00pm	2:00pm	Al-kamel Aqlan [illegible]	Lyndsey Joyce R. Ursua DMD, MSD [illegible]
19-09-2024	7 Am	12 NN	Al. kamel Aqlan [illegible]	Lyndsey Joyce R. Ursua DMD, MSD
26-09-2024	10 AM	12 N	[illegible]	Clara Nanette [illegible]
27-09 2024	1:30 PM	5 PM	Al. kamel [illegible]	Clara Nanette [illegible]
30-09 2024	7Am	12NN	Al. [illegible]	[illegible]
30-09 2024	1PM	4PM	A. k [illegible]	

Appendix HH

Receipt for SEM

ORIGINAL (Payee) — Note: Write the number and date of this receipt on the back of check or money order received.

Republic of the Philippines
UNIVERSITY OF THE PHILIPPINES DILIMAN
Diliman, Quezon City
TIN - 000-864-006-002
4092593

OFFICIAL RECEIPT
No. UPD- 4092593
DATE: 2024-10-08

PAYOR: QAID, AL-KAMEL AQLAN ALI MOQBEL

PARTICULARS	AMOUNT
9774247-499-439--SEM-EDX ANALYSIS--SEM imaging of samples without coating for 1.5 hours (unlimited photos per hour rate). Provided by Nanoworks Labor...	₱ 6,750.00

AMOUNT IN WORDS: Six Thousand Seven Hundred Fifty Pesos and 00/100

TOTAL — ₱ PhP 6,750.00

MODE OF PAYMENT ☐ CHECK/PMO ☐ CASH
Drawee Bank/Location ______
Check No.: ______ Date: ______

Received the amount stated above.

ROSCO, REVY RODRIGUEZ
CASHIER

ORIGINAL (Payee) — Note: Write the number and date of this receipt on the back of check or money order received.

Appendix II

Receipt for UTM

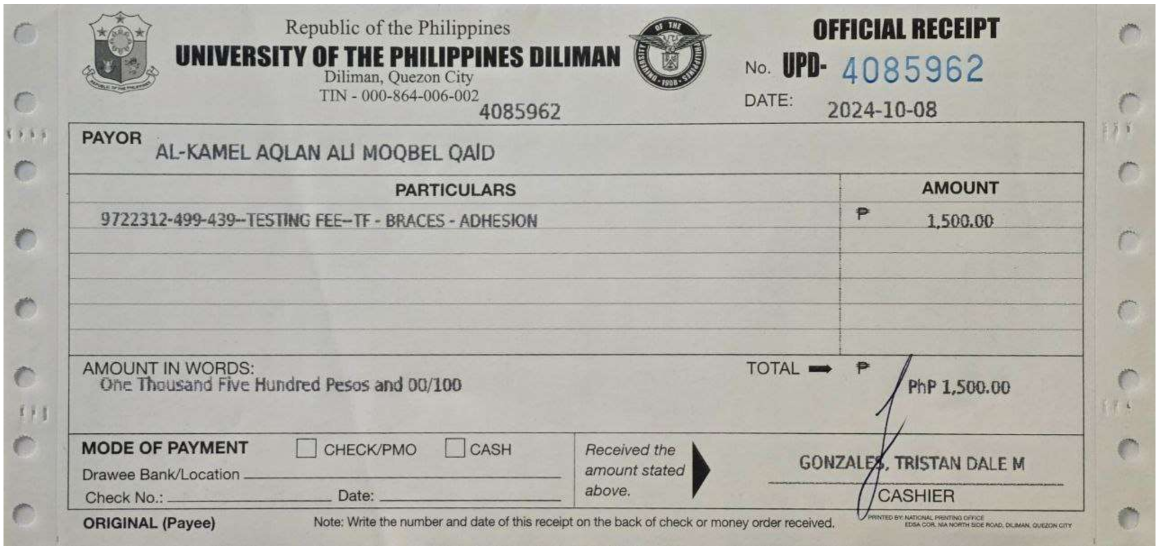

Republic of the Philippines
UNIVERSITY OF THE PHILIPPINES DILIMAN
Diliman, Quezon City
TIN - 000-864-006-002
4085962

OFFICIAL RECEIPT
No. **UPD-** 4085962
DATE: 2024-10-08

PAYOR AL-KAMEL AQLAN ALI MOQBEL QAID

PARTICULARS	AMOUNT
9722312-499-439--TESTING FEE--TF - BRACES - ADHESION	₱ 1,500.00

AMOUNT IN WORDS:
One Thousand Five Hundred Pesos and 00/100

TOTAL ➡ ₱ PhP 1,500.00

MODE OF PAYMENT ☐ CHECK/PMO ☐ CASH
Drawee Bank/Location
Check No.: Date:

Received the amount stated above.

GONZALES, TRISTAN DALE M
CASHIER

ORIGINAL (Payee)

Note: Write the number and date of this receipt on the back of check or money order received.

PRINTED BY: NATIONAL PRINTING OFFICE EDSA COR. NIA NORTH SIDE ROAD, DILIMAN, QUEZON CITY

Appendix JJ

Gantt Chart

General		
Brainstorm of Research Ideas	02-Mar-2024	18-Apr-2024
Title Proposal	19-Mar-2024	
**Research**		
Materials collection	20-Mar-2024	25-Mar-2024
Organizing Review of Related Literature and Studies	26-Mar-202	02-Aprl-2024
Reading and analysis	05-Apr-2024	10-Apr-2024
**Writing**		
Chapter 1 Completion	11- Apr -2024	14- Apr -2024
Chapter 2 Organizing Related Literatures and Studies	15- Apr -2024	19- Apr -2024
Chapter 3 Methodology	20- Apr -2024	25-Apr-2024
Fabricating a Prototype of Materials for Methodology	27-Nov-2024	30-Apr-2024
Pay the MSD Proposal fee	06-May-202	
Accomplished oral defense application form	01-May-2024	02-May-2024
Submission of Manuscript Chapter 1-3	03-May-2024	06-May-2024
Proposal defense	17-May-2024	
Gathering of materials	01-Sep-2024	04-Sep-2024
Preparation of Materials for Experiment	05-Sep-2024	06-Sep-2024
Experimentation Schedule	06-Sep-2024	03-Oct-2024
Release of Results	09-Oct-2024	
Certificates Release	10-Oct-2024	
**Editing**		
Initial review	15-Oct-2024	23-Oct-2024
Peer review	06-Nov-2024	13-Dec-2024

Appendix KK

Budget Estimation

Expenses Category	Cost
Estimate of Expenses Research Instruments	PHP 68000
Laboratory Expenses	PHP 12000
Miscellaneous	PHP 13000
Proposal defense	PHP 6300
Final defense	PHP 16570
Bookbinding	PHP 5000
Total Estimate	PHP 120870

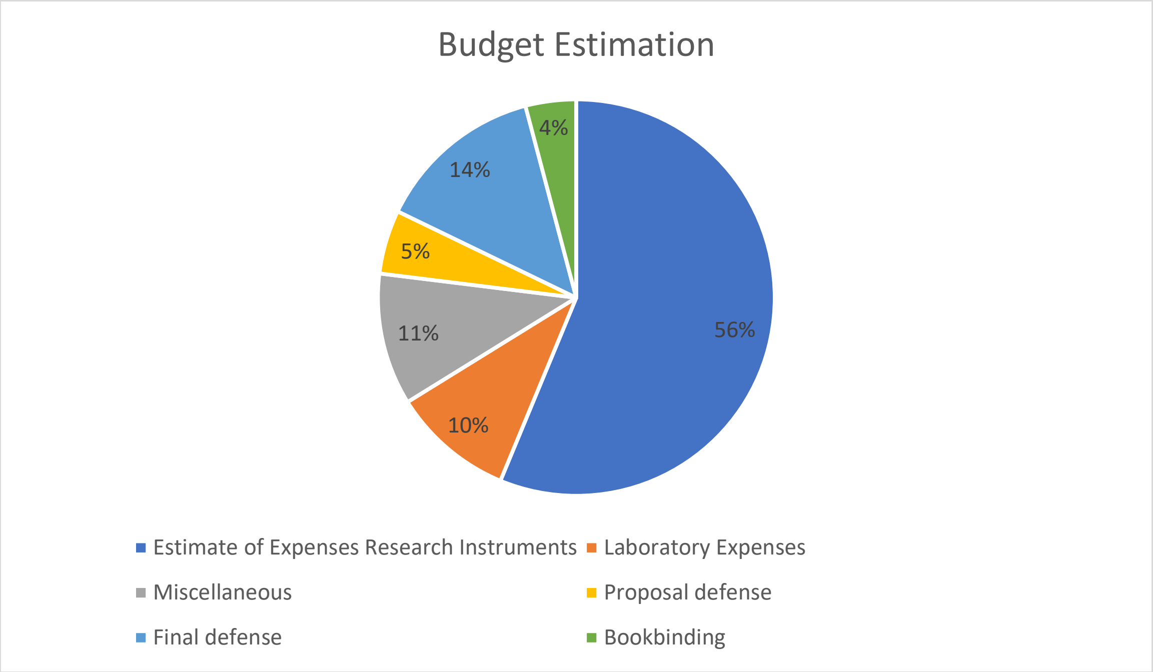

Figure 2: Budget Estimation

Appendix LL

Turnitin Similarity Test Results

Similarity Report

PAPER NAME
AL-KAMEL, AQLAN ALI MOQBEL QAID1.docx

AUTHOR
-

WORD COUNT
10453 Words

CHARACTER COUNT
60017 Characters

PAGE COUNT
62 Pages

FILE SIZE
70.4KB

SUBMISSION DATE
Oct 21, 2024 2:31 AM GMT+8

REPORT DATE
Oct 21, 2024 2:32 AM GMT+8

22% Overall Similarity

The combined total of all matches, including overlapping sources, for each database.

- 15% Internet database
- 12% Publications database
- 16% Submitted Works database

Appendix MM

CERTIFICATE OF PROOFREADING

CERTIFICATE OF PROOFREADING

This certifies that the thesis of Aqlan Ali Moqbel Qaid Alkamel titled "Comparison of Shear Bond Strength of New and Rebonded Ceramic Brackets with and without Hydrofluoric Acid Conditioning" has been edited and proofread and is hereby turned over to College of Dentistry for approval.

Shirchie P. Galvan, DMD, MSO, PhD
Language Editor
Date: November 20, 2024

Appendix NN

Receipts of materials

Receipt of monocrystalline ceramic bracket

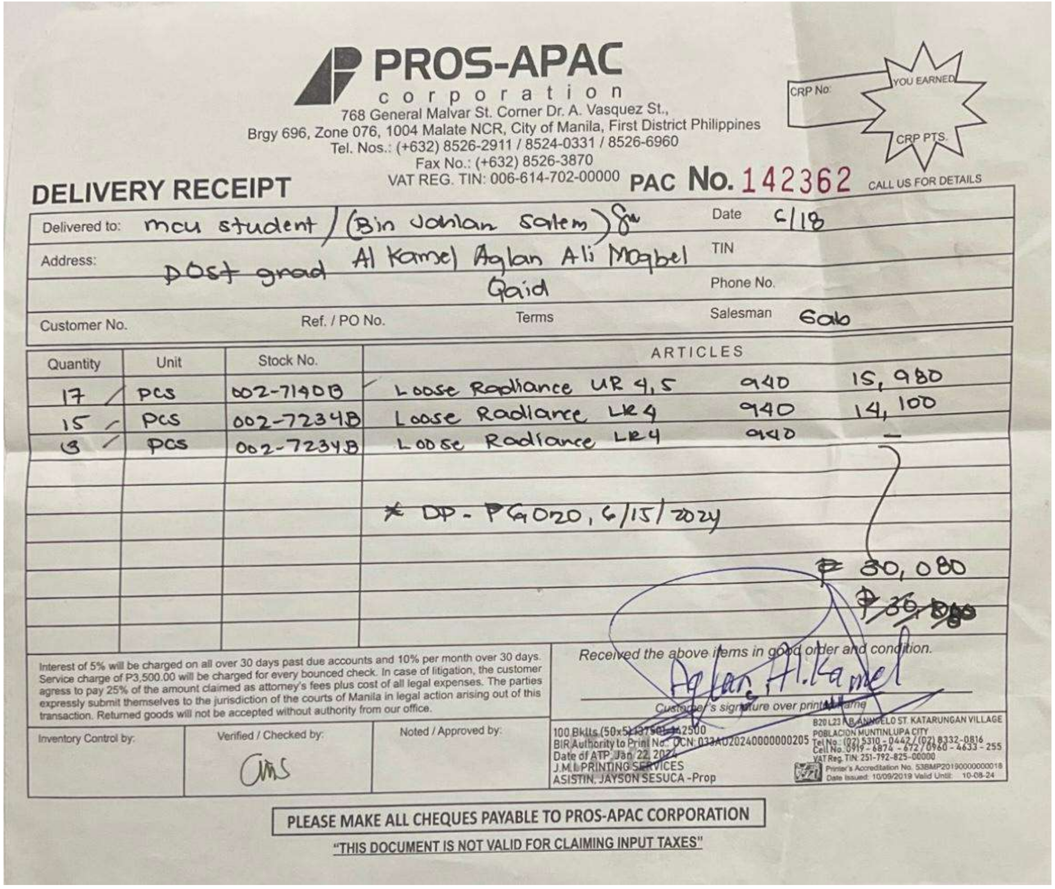

PROS-APAC corporation
768 General Malvar St. Corner Dr. A. Vasquez St.,
Brgy 696, Zone 076, 1004 Malate NCR, City of Manila, First District Philippines
Tel. Nos.: (+632) 8526-2911 / 8524-0331 / 8526-6960
Fax No.: (+632) 8526-3870
VAT REG. TIN: 006-614-702-00000

CRP No:
YOU EARNED
CRP PTS.
CALL US FOR DETAILS

DELIVERY RECEIPT — PAC No. 142362

Delivered to: mcu student / (Bin Jahlan Salem)
Date: 6/18
Address: post grad — Al Kamel Aglan Ali Moqbel Qaid
TIN
Phone No.
Customer No.
Ref. / PO No.
Terms
Salesman: Gab

Quantity	Unit	Stock No.	ARTICLES		
17	PCS	002-7140B	Loose Radiance UR 4,5	940	15,980
15	PCS	002-7234B	Loose Radiance LR 4	940	14,100
3	PCS	002-7234B	Loose Radiance LR4	940	–
			* DP - PG020, 6/15/2024		
					₱ 30,080
					~~₱ 30,080~~

Interest of 5% will be charged on all over 30 days past due accounts and 10% per month over 30 days. Service charge of P3,500.00 will be charged for every bounced check. In case of litigation, the customer agress to pay 25% of the amount claimed as attorney's fees plus cost of all legal expenses. The parties expressly submit themselves to the jurisdiction of the courts of Manila in legal action arising out of this transaction. Returned goods will not be accepted without authority from our office.

Received the above items in good order and condition.
Aglan Al-Kamel
Customer's signature over printed name

Inventory Control by:
Verified / Checked by: [signature]
Noted / Approved by:

100 Bklts (50x5) 137501-142500
BIR Authority to Print No.: OCN: 033AU20240000000205
Date of ATP: Jan 22, 2024
J.M.I. PRINTING SERVICES
ASISTIN, JAYSON SESUCA -Prop
B20 L23 F.B. ANGELO ST. KATARUNGAN VILLAGE POBLACION MUNTINLUPA CITY
Tel No.: (02) 5310 - 0442 / (02) 8332-0816
Cell No. 0919 - 6874 - 672 / 0960 - 4633 - 255
VAT Reg. TIN: 251-792-825-00000
Printer's Accreditation No. 53BMP2019000000018
Date Issued: 10/09/2019 Valid Until: 10-08-24

PLEASE MAKE ALL CHEQUES PAYABLE TO PROS-APAC CORPORATION

"THIS DOCUMENT IS NOT VALID FOR CLAIMING INPUT TAXES"

Receipt of the 3M composite resin with its bond

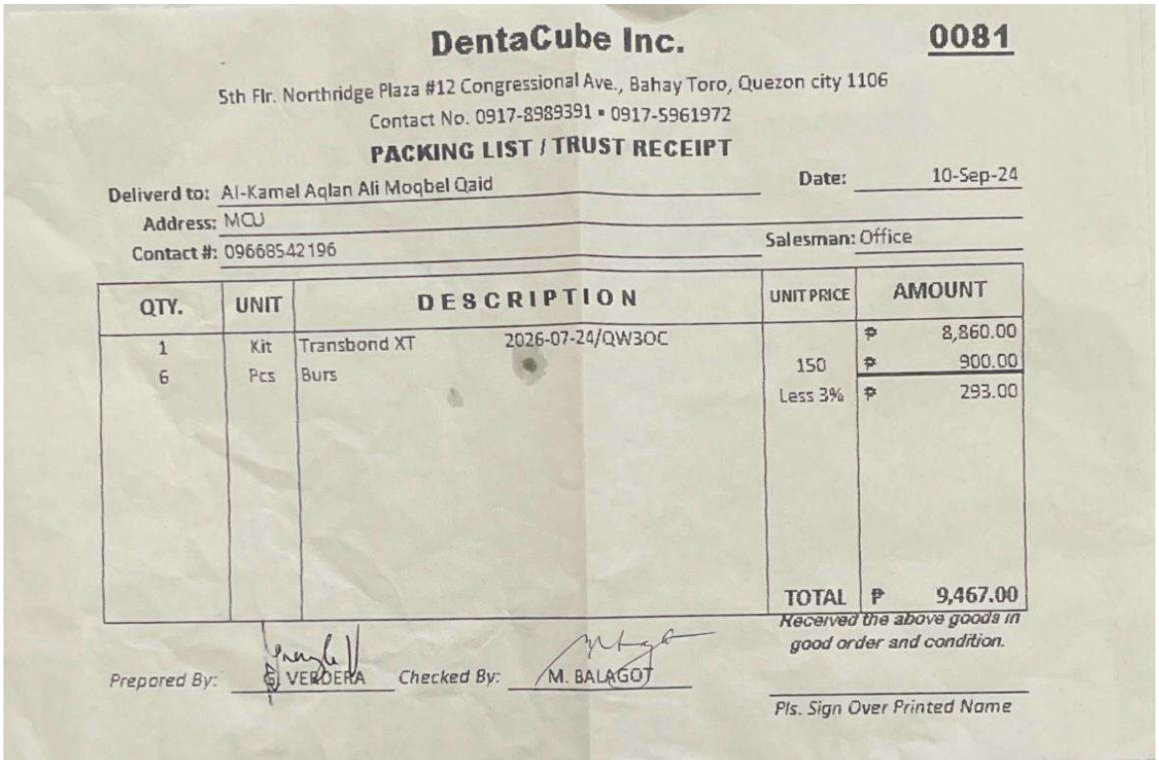

DentaCube Inc. **0081**

5th Flr. Northridge Plaza #12 Congressional Ave., Bahay Toro, Quezon city 1106
Contact No. 0917-8989391 • 0917-5961972

PACKING LIST / TRUST RECEIPT

Deliverd to: Al-Kamel Aqlan Ali Moqbel Qaid Date: 10-Sep-24
Address: MCU
Contact #: 09668542196 Salesman: Office

QTY.	UNIT	DESCRIPTION	UNIT PRICE	AMOUNT
1	Kit	Transbond XT 2026-07-24/QW3OC		₱ 8,860.00
6	Pcs	Burs	150	₱ 900.00
			Less 3%	₱ 293.00
			TOTAL	₱ 9,467.00

Received the above goods in good order and condition.

Prepared By: G. VERDERA Checked By: M. BALAGOT

Pls. Sign Over Printed Name

Receipts of Aluminum oxide 50 microns

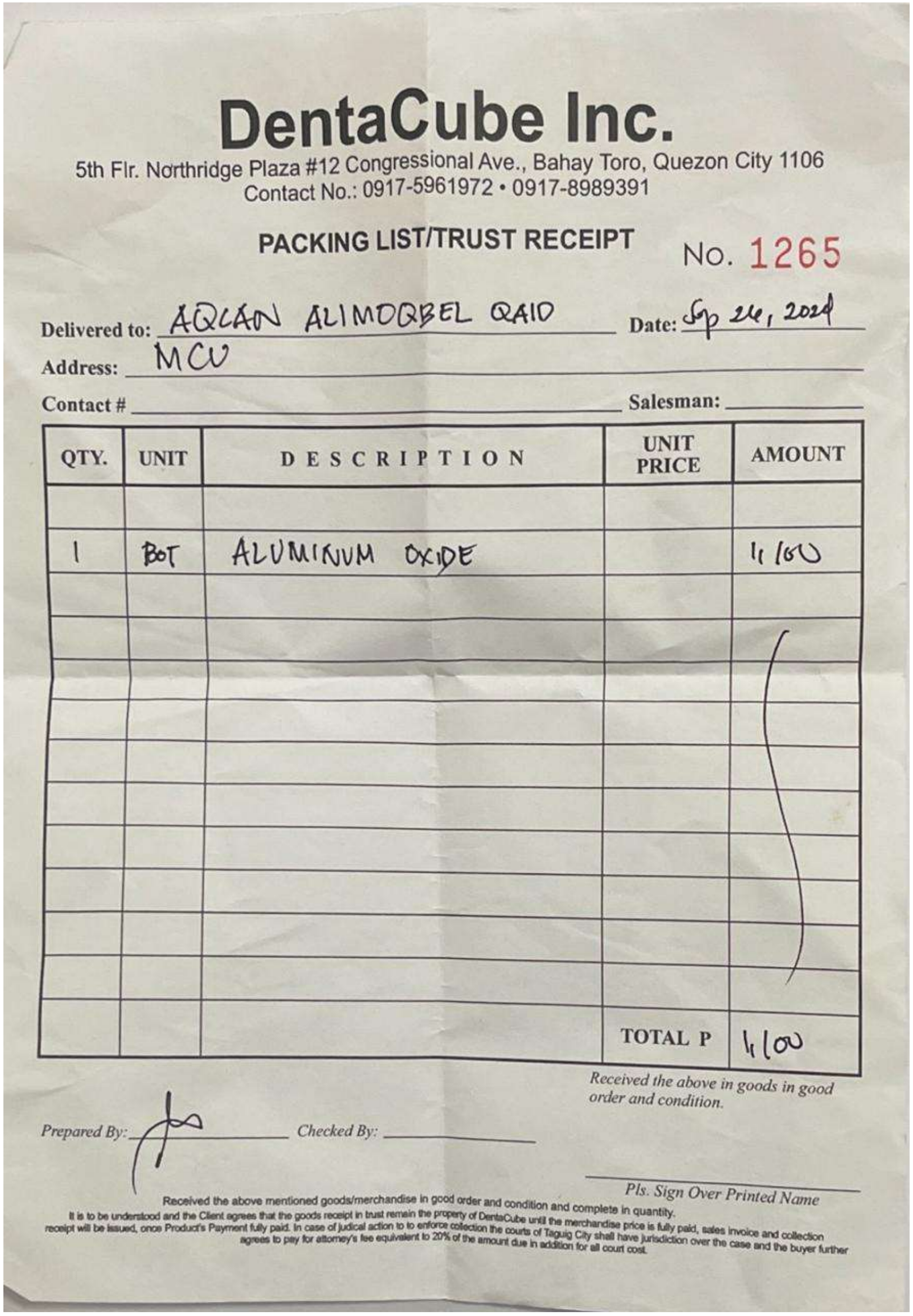

DentaCube Inc.

5th Flr. Northridge Plaza #12 Congressional Ave., Bahay Toro, Quezon City 1106
Contact No.: 0917-5961972 • 0917-8989391

PACKING LIST/TRUST RECEIPT No. 1265

Delivered to: AQLAN ALIMOQBEL QAID Date: Sep 24, 2024
Address: MCU
Contact #: Salesman:

QTY.	UNIT	DESCRIPTION	UNIT PRICE	AMOUNT
1	BOT	ALUMINUM OXIDE		1,100
			TOTAL P	1,100

Received the above in goods in good order and condition.

Prepared By: ______ *Checked By:* ______

Pls. Sign Over Printed Name

Received the above mentioned goods/merchandise in good order and condition and complete in quantity.
It is to be understood and the Client agrees that the goods receipt in trust remain the property of DentaCube until the merchandise price is fully paid, sales invoice and collection receipt will be issued, once Product's Payment fully paid. In case of judical action to to enforce collection the courts of Taguig City shall have jurisdiction over the case and the buyer further agrees to pay for attorney's fee equivalent to 20% of the amount due in addition for all court cost.

Receipt of Hydrofluoric acid 9.5% with its primer and phosphoric acid 37%

OTANER DENTAL & MEDICAL DISTRIBUTION, INC.

otaner

Rm 610-A 6th Flr Dona Felisa SyJuco Bldg Remedios St. Cor Taft Ave
Barangay 693 Zone 075 1004 Malate Ncr, City of Manila, First District Philippines
Tel. Nos.: 8 354-2458 / 8 526-0896
VAT Reg. TIN: 009-271-384-00000

DELIVERY RECEIPT No.: 79728

Delivered to: MCU STUDENT / Dr. Aqlan Ali Moqbel Qaid Alkamel		Date	September 13, 2024
Address: 90 V. MARIANO, GRACE PARK EAST CALOOCAN METRO MANILA		TIN	
		Phone No.	9668542196
Customer No. TR05418	Ref. / PO No. SSH000007814 Terms 30D	Salesman	JAN VINI QUERO

Quantity	Unit	Stock No.	ARTICLES		
1	PIECE	B 2221P 2400003100	Porcelain Primer 10ml 04/01/26	2,000.00	2,000.00
1	PIECE	E 5707P 2400001940	9 5% Porcelain Etch Refill pkg 03/11/27	1,800.00	1,800.00
1	SYRINGE	E 5623EB 2400000005	Etch 37 37% w/ BAC Syringe 10/02/26	800.00	800.00
			NAME: Aqlan Ali Moqbel Qaid Alkamel		
			Gross Amount:		4,600.00
			Redeemed Points:		
			Net Amount:		4,600.00

Interest of 5% will be charged on all over 30 days past due accounts and 10% per month over 30 days. Service charge of P3,500.00 will be charged for every bounced check. In case of litigation, the customer agress to pay 25% of the amount claimed as attorney's fees plus cost of all legal expenses. The parties expressly submit themselves to the jurisdiction of the courts of Manila in legal action arising out of this transaction. Returned goods will not be accepted without authority from our office.

Received the above items in good order and condition.

Customer's signature over printed name

Prepared by:	Checked by:	Released by:

200 Bklts (50x5) 71001-81000
BIR Authority to Print No.: OCN 033AU2024000000431
Date of ATP : Feb 8, 2024
J.M.I. PRINTING SERVICES
ASISTIN, JAYSON SESUCA -Prop

B20 [illegible] ST. KATARUNGAN VILLAGE POBLACION MUNTINLUPA CITY
Tel No. [illegible] 0919-6874672
VAT Reg. TIN: 251-792-825-00000
Printer's Accreditation No. 53BMP20190000000018
Date Issued: 10/09/2019 Valid Until: 10-08-24

PLEASE MAKE ALL CHECKS PAYABLE TO OTANER DENTAL & MEDICAL DISTRIBUTION, INC.

"THIS DOCUMENT IS NOT VALID FOR CLAIMING INPUT TAXES"

Appendix OO

Curriculum Vitae

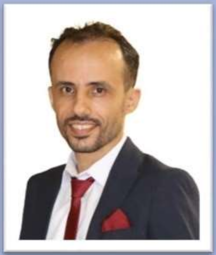

Aqlan Ali Moqbel Qaid Al-Kamel, BDS

PERSONAL INFORMATION

Aqlan Ali Moqbel Qaid Al-Kamel

Yemen Taiz, 26th September street
+967-4-253028 (+)967-770245745
aqlan.alkamel@gmail.com

Marital Status: Married
Sex: Male | Date of birth: 02-03-1988 | Place of birth: Taiz-Yemen
Nationality: Yemeni

CLINICAL SKILLS

• Orthodontist practitioner as (fixed appliance, functional appliance, preventive orthodontics, removable appliance).
• General Dentist practitioner as (endodontic treatment, scaling, fixed prosthetic…etc)
• Good dealing with patient communications.

COMPUTER SKILLS

• SPSS statistics analysis
• Good command of Microsoft Office™ tools.
• Good command of Computer Maintenance.

LANGUAGES

Mother tongue: Arabic
Another language: English (Very Good)

CAREER OBJECTIVE

I am seeking enrolment as a master student in the clinical science (orthodontics), currently pursuing a Master's degree at Manila Central University, Philippines, specializing in orthodontics.

EMPLOYMENT

- Owner and Operator - Private Dental Clinic, Taiz City, Yemen September 2017 – Until departure for studies
- Dentist - Military Hospital, Taiz, Yemen 2016 – Present
- General Dentist - Al-Muzaffar Hospital for Maternities & Infancy, Taiz, Yemen 6 November 2012 – 15 December 2014
- General Dentist - Modern Dental Centre, Taiz, Yemen August 2012 – August 2017
- Staff Member - Faculty of Dentistry, Alhekma University, Taiz, Yemen January 2015 – August 2015
- Staff Member (Part-time) - Faculty of Dentistry, Alseed University, Yemen August 2015 – Present
- Volunteer Dentist - Al-Thawra General Hospital, Taiz, Yemen 4 October 2015 – 5 December 2016

EDUCATION

- Master's Degree in Orthodontics (Final Year) - Manila Central University, Philippines Currently completing the final month of the program.
- Bachelor's Degree in Dentistry - Ibb University, Faculty of Dentistry 2011 - 2012
- Internship Certificate - Military Hospital, Taiz, Yemen 6 November 2012 - 5 November 2013
- Secondary School Graduate - Taiz Secondary School, Taiz, Yemen 2005

CONFERENCES AND COURSES ATTENDED

- Implant Training Course 8-10 January 2014
- MAKKAH Dental Conference - Makkah, Saudi Arabia 1-3 April 2014
- Third Scientific Dental Conference - Yemen Dental Association, 45th Scientific of Arab Dental Federation 18-20 December 2014
- Biostatistics and SPSS Program Course - Alhekma University, Yemen 27 December 2014 – 5 January 2015
- Workshop on Course Description for Dental Assistance Department - Taiz University, Yemen 6-9 November 2016
